# Iron overload exaggerates renal ischemia-reperfusion injury by promoting tubular cuproptosis via interrupting function of LIAS

**DOI:** 10.1016/j.redox.2025.103795

**Published:** 2025-08-05

**Authors:** Siyue Chen, Tingting Chen, Cuidi Xu, Xiaohan Yu, Junyu Shi, Cheng Yang, Tongyu Zhu

**Affiliations:** a Department of Kidney Transplantation, Zhongshan Hospital, Fudan University, China; b Shanghai Key Laboratory of Organ Transplantation, Shanghai, 200032, China; c Shanghai Medical Collage, Fudan University, Shanghai, China; d Zhangjiang Institue of Fudan University, Shanghai, China; e Department of Pharmacy, Zhongshan Hospital, Fudan University, Shanghai, China

**Keywords:** Renal ischemia-reperfusion injury, Acute kidney injury (AKI), Cuproptosis, Ferroptosis, Metal ion homeostasis, Iron overload, Copper cytotoxicity, Protein lipoylation

## Abstract

Renal ischemia-reperfusion injury (RIRI), a major contributor to acute kidney injury (AKI) and delayed graft function (DGF), is closely associated with dysregulation of metal ion homeostasis. Although copper and iron metabolism exhibit interconnected regulatory pathways, the temporal dynamics and functional interplay of these metal ions in RIRI pathogenesis remain poorly understood. Our study demonstrates that cuproptosis and ferroptosis, two distinct forms of cell death induced by metal ion overload, occur simultaneously within 6 h after reperfusion. Notably, ischemia-reperfusion injury induced iron overload significantly sensitizes renal tubular cells to copper-mediated cytotoxicity. Mechanistic investigations demonstrate that hypoxia-reoxygenation triggers Fe (II) accumulation, which subsequently downregulates [4Fe–4S] cluster assembly proteins. This impairment directly compromises the structural integrity of the cuproptosis-regulating protein LIAS by inducing [4Fe–4S] cluster loss, ultimately leading to defective protein lipoylation that drives cuproptosis progression. Crucially, these pathological effects can be attenuated through either overexpression of [4Fe–4S] cluster assembly machinery or therapeutic application of iron-chelating agents. Our findings establish a novel iron-copper crosstalk mechanism in RIRI pathophysiology and propose targeted strategies focusing on [4Fe–4S] cluster homeostasis and iron chelation for clinical intervention.

## 1 Introduction

Renal ischemia-reperfusion injury (RIRI) constitutes a critical pathway to acute kidney injury (AKI) and delayed graft function (DGF) in transplantation settings [1,2]. Despite its clinical prevalence, therapeutic options remain limited due to incomplete understanding of tubular cell death mechanisms. Emerging evidence identifies metal ion dyshomeostasis as a central pathological driver in myocardial and cerebral ischemia models, where metal overload exacerbates injury through ferroptosis and cuproptosis, two regulated cell death pathways mechanistically linked to iron-dependent lipid peroxidation and copper-mediated proteotoxic stress, respectively [[3], [4], [5], [6]].

The interplay between iron and copper homeostasis is governed by three principal physiological mechanisms: (1) Redox coupling of ferrous Fe (II) and cuprous Cu (I) ions mediated by specific proteins including Six-Transmembrane Epithelial Antigen of the Prostate (STEAP); (2) Functional coordination within mitochondrial electron transport chain complexes, where copper ions mediate cytochrome *c* oxidase activity while iron ions contribute to iron-sulfur cluster biogenesis in succinate dehydrogenase; (3) Complementary Fenton catalysis, where iron initiates •OH formation and copper accelerates radical chain reactions via distinct electron transfer pathways [[7], [8], [9], [10], [11]]. This metabolic crosstalk suggests pathophysiological interactions, as evidenced in hepatocellular carcinoma where copper dysregulation exacerbates ferroptosis through impaired iron homeostasis [12]. However, critical uncertainties persist regarding RIRI-specific mechanisms, including (1) whether ferroptosis and cuproptosis occur simultaneously during RIRI progression; (2) how iron-copper homeostasis mediates their crosstalk; (3) how systemic and tissue-specific changes in metal homeostasis evolve during RIRI; (4) whether therapeutic targeting of this metal regulatory axis shows preclinical promise.

This knowledge gap is particularly significant given the kidney's unique metal handling requirements. Proximal tubules exhibit high metabolic demand for oxidative phosphorylation closely correlated to [4Fe–4S] cluster proteins like lipoic acid synthase (LIAS), which requires coordinated iron-copper regulation for proper function [[13], [14], [15]]. LIAS catalyzes the synthesis of lipoic acid, a critical cofactor for mitochondrial enzyme complexes, and its activity is tightly linked to the integrity of its [4Fe–4S] cluster [[16], [17], [18]]. Dysregulation of iron homeostasis can impair the biosynthesis and assembly of iron-sulfur cluster proteins [19]. This disruption could further sensitize cells to copper toxicity, as LIAS-generated lipoylated proteins are key mediators of cuproptosis, a copper-dependent cell death pathway [20,21]. We therefore hypothesize that RIRI-induced iron overload may predispose tubular cells to copper toxicity by disrupting [4Fe–4S] cluster assembly and LIAS-mediated metabolic processes.

Here, we investigated the spatiotemporal coordination between cuproptosis and ferroptosis in a murine RIRI model. Our data demonstrate that both death modalities manifest within 6 h post-reperfusion, with iron overload acting as a key sensitizer for copper-mediated cytotoxicity. Mechanistically, hypoxia-reoxygenation-induced Fe (II) accumulation disrupts [4Fe–4S] cluster biogenesis, leading to structural destabilization of LIAS and subsequent failure in protein lipoylation. Importantly, therapeutic interventions targeting either Fe–S cluster restoration or iron chelation significantly attenuated tubular cell death. These findings not only establish a novel iron-copper regulatory axis in RIRI but also provide actionable targets for clinical intervention.

## 2 Results

### 2.1 Ferroptosis and cuproptosis occurred simultaneously after RIRI

To investigate the interplay between iron and copper metabolism in renal ischemia-reperfusion injury (RIRI), we first analyzed clinical data from patients with delayed graft function (DGF) following kidney transplantation. The results revealed that DGF patients exhibited significantly lower serum iron levels and higher serum copper levels on postoperative day 2 and day 30 compared to non-DGF patients and a positive correlation with serum creatinine levels in these patients, indicating a dysregulation of iron-copper homeostasis in RIRI (Fig. 1A, S1A-B). To further explore this phenomenon, we measured the concentrations of copper and iron ions in mouse kidney tissue at 3-, 6-, 12-, 24-, and 48-h post IR surgery (Fig. 1B). The results showed that both copper and iron ion concentrations in renal tissue exhibited an initial decline followed by an increase. Notably, the peak copper ion concentration did not exceed that of the sham group. In contrast, ferrous iron [Fe(II)], which represents the primary intracellular reactive iron species, displayed progressive elevation starting at 3 h after surgery, exceeded the sham levels by 6 h, and reached peak concentration at 12 h (Fig. 1B). The serum levels of copper and iron ions in mice exhibited changes following RIRI that mirrored the trends observed in patients with DGF.

**Fig. 1:**
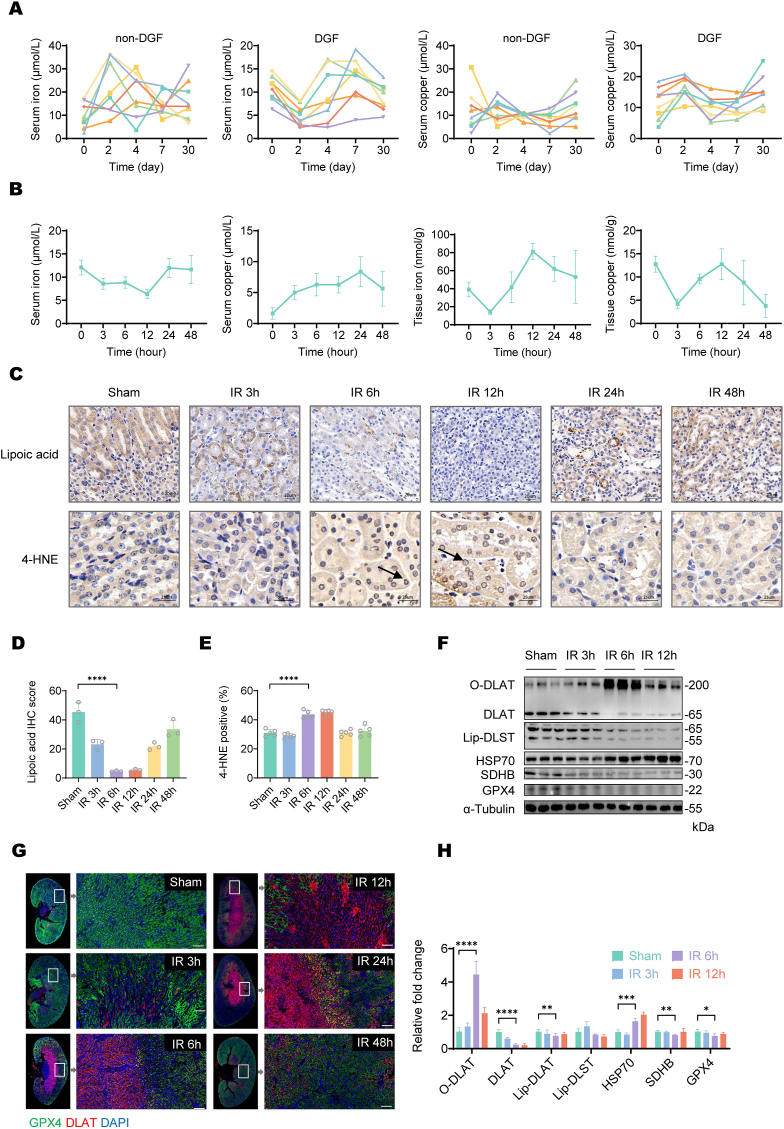
**Ferroptosis and cuproptosis occurred simultaneously after RIRI.** (A) The line graph illustrates the dynamic changes in serum iron levels or serum copper levels in patients with DGF and those non-DGF at pre-transplantation and multiple post-transplantation time points. DGF patients exhibited a distinct trend of iron characterized by an initial decrease, followed by a subsequent increase, and then a gradual decline, ultimately stabilizing over time and a distinct trend of copper characterized by an initial increase, followed by a subsequent decrease, and then a gradual rise, ultimately stabilizing over time. (B) Serum iron concentration, serum copper concentration, tissue iron concentration and tissue copper concentration of mice at different time points post RIRI. (C) 4-HNE IHC staining of mice kidney indicates the presence of lipid peroxidation. The arrows indicate lipid peroxidation around the nuclear membrane, suggesting increased cellular lipid peroxidation (Magnification: × 400, Scale bar: 25 μm). Lipoic acid IHC staining of mouse kidney tissues indicated significantly reduced lipoylation levels in the 6 h post RIRI group compared to sham controls (Magnification: × 200, Scale bar: 25 μm). (D) Quantification of Lipoic acid IHC staining score across different time point. Data are presented as mean ± SD, n = 6. (E) Quantification of positive area of 4-HNE IHC staining across different time point. Data are presented as mean ± SD, n = 6. (F) Western blot of mice kidney tissue post RIRI at different time points. α-Tublin was used as a loading control. (G) Representative IF staining of DLAT (red), GPX4(green), and DAPI (blue) in kidney from mice (Magnification: × 200, Scale bar: 25 μm). (H) Quantification of Western blot of mice kidney tissue post RIRI at different time points. The bar graph represents the average of independent experiments. Data are presented as mean ± SD, n = 6 for all groups.

Next, we investigated the occurrence of ferroptosis and cuproptosis following RIRI. IHC assays revealed increased lipid peroxidation, as evidenced by elevated 4-HNE levels at 6 h post-reperfusion (Fig. 1C–E). Concurrently, lipoic acid staining showed significantly reduced levels, which reflects impaired biosynthesis or utilization of lipoic acid, a recognized hallmark of cuproptosis (Fig. 1C and D). Western blot analysis further corroborated these findings. GPX4, a key negative regulator of ferroptosis, was significantly downregulated at 6 h post-operation (Fig. 1F). Additionally, we observed characteristic features of cuproptosis, including DLAT oligomerization, downregulation of the Fe–S cluster protein SDHB, and elevated HSP70 expression (Fig. 1F). Together, these results demonstrate the activation of both ferroptotic and cuproptotic pathways during RIRI. Immunofluorescence confirmed that GPX4 downregulation and DLAT oligomerization primarily occurred in tubular cells at 6 h post-operation (Fig. 1G). However, a notable discrepancy emerged: ferroptosis at 6 h post-operation was directly associated with intracellular ferrous ion accumulation, whereas cuproptosis occurred without requiring copper ion concentrations to exceed normal baseline levels.

To unveil the link between tubular metal ion accumulation after renal ischemia-reperfusion injury (IRI), we established an *in vitro* hypoxia-reoxygenation (H/R) model to simulate the process (Fig. 2A). ICP-MS analysis revealed increased cellular concentrations of both iron and copper ions after 6 h of reoxygenation, although the rise in copper ions was not significant (Fig. 2B). Consistent with our *in vivo* findings, markers of cuproptosis and ferroptosis were observed in the H/R model (Fig. 2C–E, S2E).

**Fig. 2:**
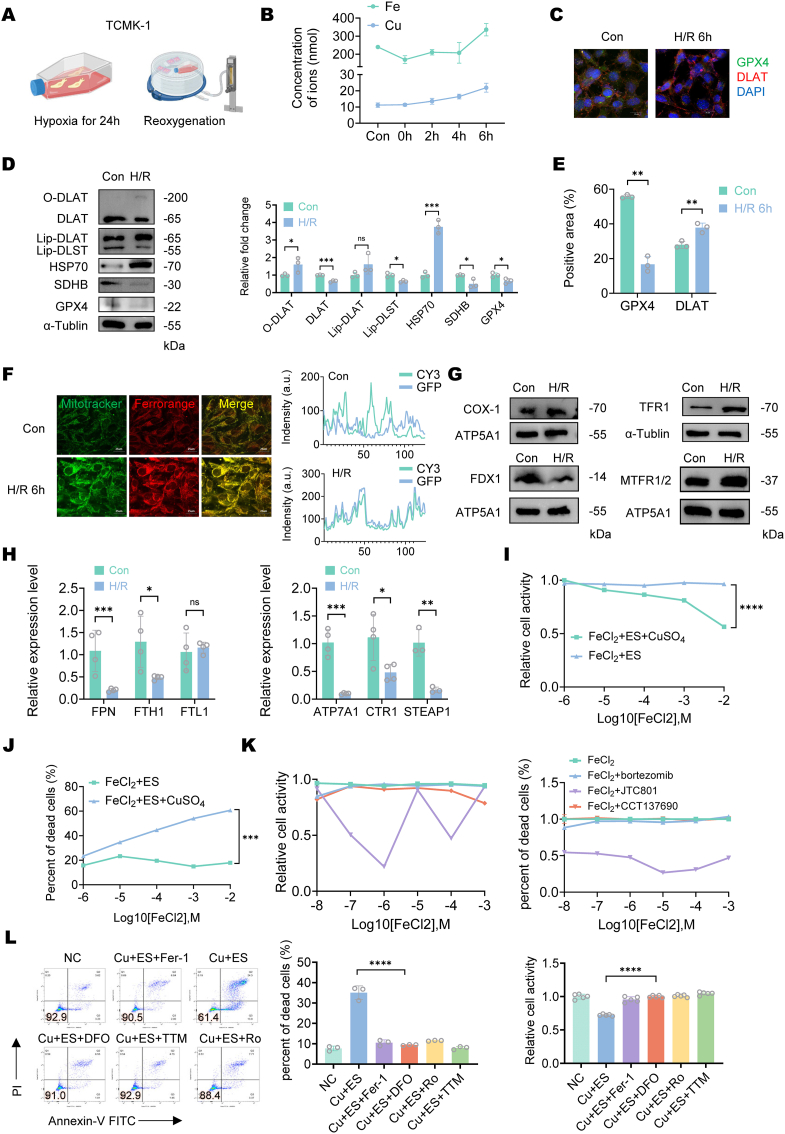
**Iron overload promotes elesclomol-Cu (II) induced cuproptosis *in vitro*.** (A) Schematic diagram of Hypoxia-Reoxygenation cell model establishment. (B) Copper and iron ion concentrations in 2 × 10^7^ TCMK-1 cells subjected to 24-h hypoxia followed by reoxygenation for 0, 2, 4, and 6 h. Ion concentrations were quantified by inductively coupled plasma mass spectrometry (ICP-MS). Data are presented as mean ± SD, n = 3. (C) Representative IF staining of DLAT (red), GPX4 (green), and DAPI (blue) of TCMK-1 cells post H/R 6 h and control group (n = 3 in each group, Magnification: × 400, Scale bar: 20 μm). (D) Western blot of TCMK-1 cells post H/R 6 h and the control group. α-Tublin was used as a loading control. The bar graph represents the average of independent experiments. Dots represent individual data points. Data are presented as mean ± SD, n = 3 for all groups. (E) Quantitative analysis of fluorescence intensity demonstrated a significant increase in DLAT aggregation (p < 0.01) and decrease in GPX4 expression (p < 0.05) compared to sham controls. Data are presented as mean ± SD, n = 3 for all groups. (F) Representative images of mitochondrial and Fe^2+^ co-staining in control and hypoxia (24 h)-reoxygenation (6 h)-treated TCMK-1 cells. Mitochondria were labeled with green fluorescence, and Fe^2+^ was detected using a red fluorescent probe. Images demonstrate mitochondrial iron accumulation following hypoxia-reoxygenation injury. Scale bar: 25 μm. The overlapping fluorescence patterns suggest a close association between mitochondrial localization and iron accumulation during H/R. (G) Western blot of mitochondria extracted from TCMK-1 cells or TCMK-1 cells post H/R 6 h and control group. ATP5A1 was used as a mitochondrial protein loading control and α-Tublin was used as a cellular protein loading control. Cox-1, representing mitochondrial copper concentration, showed no significant change (p > 0.05) following hypoxia-reoxygenation injury. FDX-1, a copper transport protein responsible for mitochondrial copper delivery, was significantly downregulated (p < 0.05) after hypoxia-reoxygenation treatment. n = 3 for all groups. (H) Real-time quantitative PCR (RT-Qpcr) analysis of metal ion regulatory genes in control and hypoxia-reoxygenation-treated cells (n = 4). mRNA levels of copper export (ATP7A) and uptake (steap1, CTR1) genes were quantified. H/R treatment significantly downregulated CTR1, ATP7A, STEAP1 expression (p < 0.05). mRNA levels of iron export (FPN) and storage (FTH, FTL) genes were quantified. H/R treatment significantly downregulated FPN expression (p < 0.01) while upregulating FTH (p < 0.05). Data are presented as mean ± SD. (I) Cell death rate of TCMK-1 cells following sequential treatment with FeCl_2_ (10 nM–1 mM, 24 h) and elesclomol-Cu (II) (10 nM, 2 h). Cell death was assessed by Annexin V and propidium iodide (PI) staining followed by flow cytometry analysis. The percentage of cell death was calculated as 100 % minus the proportion of double-negative (Annexin V^−^/PI^−^) cells. Statistical significance was determined by t-t test (n = 3). (J) Cell viability of TCMK-1 cells following sequential treatment with FeCl_2_ (10 nM–1 mM, 24 h) and elesclomol-Cu (II) (10 nM, 2 h). Viability was assessed using the CCK-8 assay (n = 5). Statistical significance was determined by t-t test (n = 3). (K) Cell viability and cell death rate of TCMK-1 cells following sequential treatment with FeCl_2_ (10 nM–1 mM, 24 h) and apoptosis inducer (D, bortezomib, 5 μm, 12 h), alkaliptosis inducer (E, JTC801, 10 μm, 12 h) or necroptosis inducer (F, CCT137690 10 μM, 12 h). (L) Cell death rate and cell viability of TCMK-1 cells treated with elesclomol-Cu (II) in the presence or absence of Ferrostatin-1 (Fer-1, 5 μM, an antioxidant), Deferoxamine (DFO, 50 μM, an iron chelator), and Rosiglitazone (Ro, 1 μM, an ACSL4 inhibitor) or tetrathiomolybdate (TTM, 50 μM, a copper chelator) for 24 h. Statistical significance was determined by one-way ANOVA (n = 3).

Since cuproptosis is driven by disruptions in the tricarboxylic acid (TCA) cycle, we further examined mitochondrial metal ion dynamics. Ferrorange and MitoTracker co-staining demonstrated that intracellular Fe (II) levels increased and accumulated in mitochondria after H/R (Fig. 2F), while COX-1, a marker of mitochondrial copper levels, remained unchanged (Fig. 2G–S2C) [22]. The detection of key metal transport proteins supported these observations. The upregulation of cellular iron uptake protein TFR1 and mitochondrial iron uptake proteins mTFR1/2, coupled with downregulation of iron efflux protein FPN, collectively promoted iron accumulation (Fig. 2G–H, S2C). However, the copper transport profile revealed a distinct pattern: although the cellular copper efflux gene ATP7A was downregulated, both the cellular copper uptake protein CTR1 and mitochondrial copper uptake protein FDX1 were also downregulated. This coordinated downregulation of both copper influx and efflux components argues against a net increase in cellular copper retention (Fig. 2G–H, S2C).

Despite the co-occurrence of ferroptosis and cuproptosis following RIRI, total cellular copper ion levels remained unchanged in our experimental model. This apparent paradox suggests that cuproptosis in this context may not depend on systemic copper overload. Based on these findings, we propose that iron overload potentiates cellular susceptibility to cuproptosis by disrupting iron-copper homeostasis and amplifying mitochondrial metal toxicity.

### 2.2 Iron overload promotes elesclomol-Cu induced cuproptosis *in vitro*

To investigate the interplay between iron overload and cuproptosis susceptibility, renal tubular epithelial cells were cultured in media with FeCl_2_ (10 nM–1 mM) and treated with equal concentrations of the cuproptosis-inducing agent elesclomol-Cu (II) **(**ES-Cu (II)) (10 nM) or its control compound ES (10 nM). The results demonstrated that with increasing concentrations of FeCl_2_, ES-Cu treatment led to a significant decline in cell viability (Fig. 2I), accompanied by progressively escalating cell death (Fig. 2J–S2D). In contrast, the proportion of dead cells and viability remained unchanged in the control group treated with ES alone (Fig. 2I–J, S2D). As a control, a concentration gradient of FeCl_2_ failed to enhance bortezomib-induced apoptosis, CCT137690-triggered necroptosis, or JTC801-initiated alkaliptosis, indicating that iron overload does not exert a nonspecific cytotoxic effect on these forms of cell death (Fig. 2K).

Since iron overload can directly lead to ferroptosis, we hypothesized that the increased sensitivity of cells to cuproptosis might be related to the activation of ferroptosis-related pathways. To test this, we pretreated cells with three different ferroptosis inhibitors: Ferrostatin-1 (Fer-1, 5 μM, an antioxidant), Deferoxamine (DFO, 50 μM, an iron chelator), and Rosiglitazone (Ro, 1 μM, an ACSL4 inhibitor), followed by exposure to ES-Cu (II) to induce cuproptosis. Our results showed that all three ferroptosis inhibitors provided some protective effects against ES-Cu (II)-induced cuproptosis, though their efficacy was weaker than that of the copper chelator tetrathiomolybdate (TTM, 50 μM). Among them, the DFO pretreatment group exhibited the lowest proportion of cell death and the highest cell activity. These findings suggest that activation of the ferroptosis pathway promotes cuproptosis, and that reducing iron ion accumulation can mitigate cuproptosis (Fig. 2L).

Previous studies have confirmed that depleting intracellular glutathione (GSH) may be a key factor in triggering cuproptosis, prompting us to further investigate this mechanism. By supplementing with the antioxidant GSH under iron-overloaded conditions, we observed that although increased GSH levels effectively reduced reactive oxygen species (ROS) generation (Fig. S2C), iron overload-induced cuproptosis was not fully rescued (Fig. S2D). These results suggest that iron overload-driven cuproptosis may involve additional regulatory factors independent of oxidative stress.

### 2.3 Iron overload promotes elesclomol-Cu induced cuproptosis *in vivo*

To further elucidate the role of cuproptosis in kidney injuries, we conducted *in vivo* experiments by intraperitoneally administering ES-Cu to mice (Fig. 3A). Three days post-injection, the animals developed significant renal injury, as evidenced by elevated serum creatinine levels and histopathological changes including tubular damage and crescent formation (Fig. 3B–E). Co-administration of ES-Cu and DFO attenuated renal injury, with no crescent formation observed and reduced serum creatinine levels (Fig. S3H). We further validated whether iron overload promotes cuproptosis *in vivo*. In previous experiments, we observed that an increase in renal iron levels was accompanied by a decrease in serum iron (Fig. 1B). Therefore, to investigate the role of iron overload in promoting cuproptosis, we avoided inducing systemic iron overload through a high-iron diet. Instead, we employed an adeno-associated virus (AAV) to deliver a shRNA coding vector under the control of the tubular-specific Ksp promoter, which enabled conditional knockdown of FPN, the iron export transporter, specifically in renal tubular epithelial cells, thereby achieving localized iron overload in the renal tubules (Fig. 3F).

**Fig. 3:**
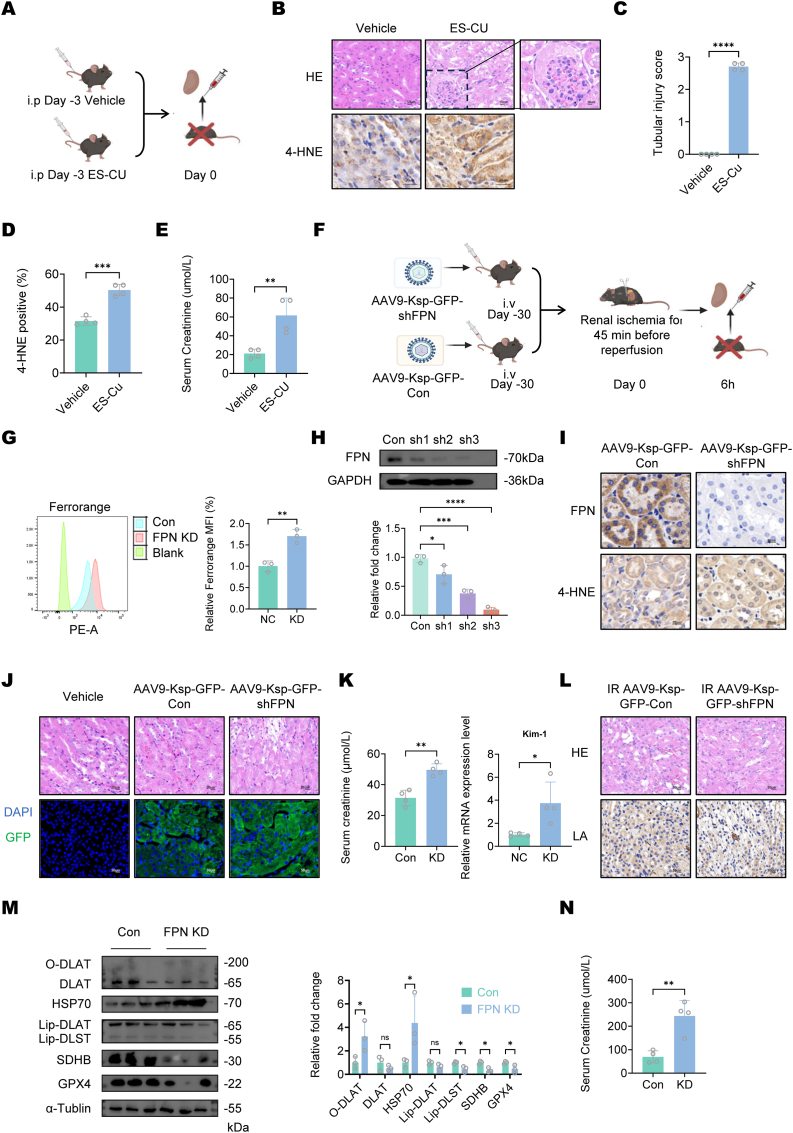
**Iron overload promotes elesclomol-Cu (II) induced cuproptosis *in vivo*.** (A) Schematic Representation of Experimental Timeline and Treatment Groups. The Cu-Elesclomol complex (ES-Cu) was prepared by mixing Elesclomol and CuSO_4_ at equimolar concentrations, mice were administered 0.1 mL of the ES-Cu complex via intraperitoneal injection at a dose of 100 μmol/kg body weight 3 days before sacrificed (based on Elesclomol concentration). (B) Representative H&E staining and 4-HNE IHC staining of renal tissues from different treatment groups. (Magnification: × 200, Scale bar: 50 μm). Images show significant tubular injury and the formation of crescents, indicative of severe renal damage with increasing lipid peroxidation. (C) Tubular injury score of mice in different group were shown in bar chart. Data are presented as mean ± SD, n = 4.(Magnification: × 200, Scale bar: 50 μm) (D) Quantification of positive area of 4-HNE IHC staining across different groups. Data are presented as mean ± SD, n = 4. (E) Serum creatinine of mice in different groups. Data are presented as mean ± SD, n = 4. (F) Schematic diagram of animal model establishment and sample collection. Tissue collection was performed either directly 30 days after tail vein injection or following unilateral nephrectomy with contralateral renal ischemia-reperfusion injury (RIRI) induction (6 h post-reperfusion). (G) Fe^2+^ levels in control and FPN-knockdown TCMK-1 cells were assessed using the Ferrorange probe. Mean fluorescence intensity (MFI), representing Fe^2+^ concentration, was quantified by flow cytometry. Data are presented as mean ± SD, n = 3. (H) Western blot analysis at 60 h post-transfection confirmed significant FPN downregulation by all three shRNAs compared to scrambled controls. Data are presented as mean ± SD, n = 3. (I) IHC staining of FPN confirmed successful FPN knockdown in AAV9-transduced tissues. 4-HNE IHC staining of mice kidney indicates the presence of lipid peroxidation. (Magnification: × 400, Scale bar: 50 μm) (J) AAV carrying GFP labeled vectors were injected, and transduction efficiency was assessed by GFP fluorescence (green) and DAPI staining (blue). Representative images show GFP expression specifically in tubular structures of AAV-injected tissues, confirming successful transduction. Scale bar: 50 μm. Representative H&E staining images of renal tissues from control and FPN-knockdown mice show tubular injury in KD mice. H&E staining showed tubular edema and nuclear pyknosis in some tubular cells. (K) Serum creatinine and KIM-1 expression level of mice in control group and FPN knock down group. KIM-1 expression level was assessed by RT-qPCR analysis. Data are presented as mean ± SD, n = 4. (L) Representative H&E and lipoic acid (IHC) staining of renal tissues from control and FPN-knockdown mice subjected to unilateral nephrectomy and contralateral renal ischemia-reperfusion injury (45 min ischemia). Scale bars: 50 μm. (M) Western blot analysis of renal tissues from control and FPN-knockdown mice. Dots represent individual data points. Data are presented as mean ± SD, n = 4 for all groups. (N) Serum creatinine of mice in control group and FPN knock down group subjected to unilateral nephrectomy and contralateral renal ischemia-reperfusion injury (45 min ischemia). Data are presented as mean ± SD, n = 4 for all groups.

We validated that knockdown of FPN effectively increases intracellular iron concentrations in renal tubular cells, establishing a mechanistic foundation for subsequent *in vivo* studies (Fig. 3G). The infection efficiency of AAV and the knockdown efficacy were confirmed by GFP autofluorescence detection and FPN IHC staining (Fig. 3H–J, S3A). Increased renal iron concentration correlated with elevated lipid peroxidation (Fig. 3I–S3F), while copper levels remained unchanged (Fig. S3G). Intriguingly, IHC staining revealed reduced lipoic acid signal intensity following FPN knockdown (Fig. S3C and S3E), and Western blot analysis demonstrated increased DLAT oligomerization, concomitant downregulation of the Fe–S cluster protein SDHB, decreased GPX4 level and elevated HSP70 expression indicating activated ferroptosis and cuproptosis (Fig. 3M). HE staining showed tubular edema and nuclear pyknosis in some tubular cells (Fig. 3J S3B). Although serum creatinine levels exhibited a mild elevation, increased expression of the tubular injury marker KIM-1 indicated tubular damage (Fig. 3K). These findings collectively suggest that localized iron overload in renal tubules may concurrently induce cuproptosis and ferroptosis in tubular epithelial cells, even in the absence of overt systemic renal dysfunction. Then, we performed IR surgery on FPN knock down (KD) and control mice, results showed that the FPN KD group exhibited more severe damage compared to the control group, with a decrease in lipoic acid levels (Fig. 3L and N, S3D).

### 2.4 Iron overload suppresses mitochondrial [4Fe–4S] cluster assembly in tubular cells

Given the mitochondrial roles of cuproptosis-related proteins in the TCA cycle and previous evidence of iron overload-induced mitochondrial dysfunction, as evidenced by reduced mitochondrial membrane potential (ΔΨm) (Fig. S4A), we conducted quantitative proteomic analysis of mitochondria isolated from control and iron-overloaded TCMK-1 cells. Prior to analysis, we validated our iron overload model by first determining the optimal FeCl_2_ concentration capable of inducing both ferroptosis and cuproptosis, which elicited lipid peroxidation levels and expression patterns of HSP70, Fe–S cluster protein SDHB, and lipoylated proteins comparable to hypoxia-reoxygenation injury, thereby effectively recapitulating the pathophysiological iron dysregulation observed during H/R conditions. (Fig. 4A–D).

**Fig. 4:**
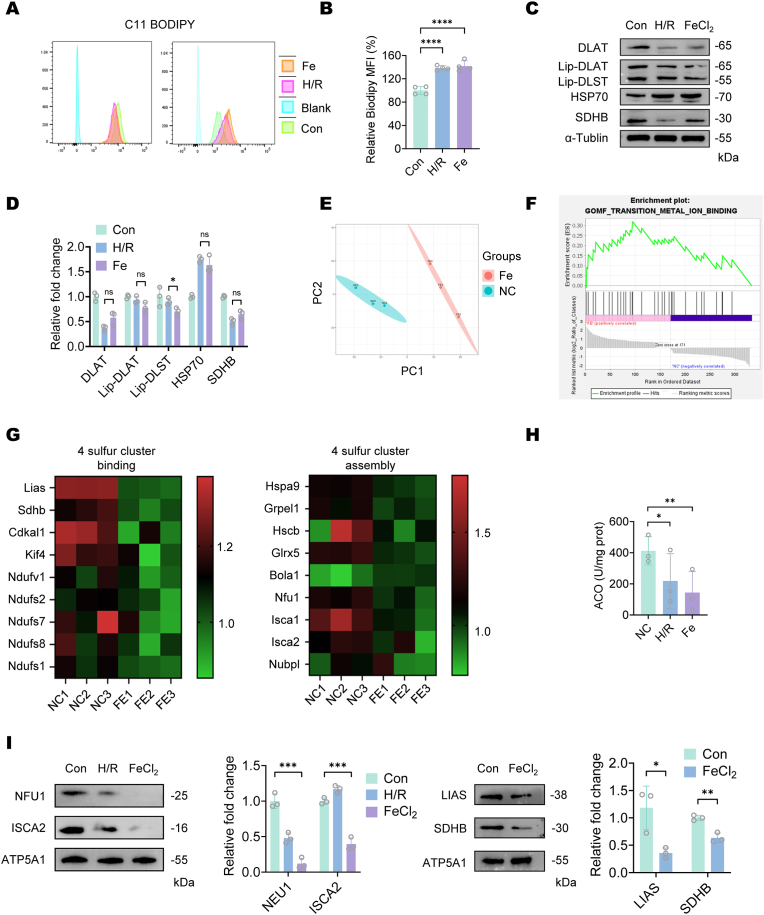
**Iron overload suppresses mitochondrial [4Fe–4S] cluster assembly in tubular cells.** (A) Lipid peroxidation levels assessed by C11-BODIPY staining and flow cytometry analysis. (B) Mean fluorescence intensity (MFI) was quantified in control and experimental groups. Data are presented as mean ± SD, n = 3. (C) Western blot analysis of TCMK-1 cells post H/R 6 h, treated with FeCl_2_ (100 μM,24 h) and control group. (D) Quantification of Western blot of different groups. The bar graph represents the average of independent experiments. Data are presented as mean ± SD, n = 3 for all groups. (E) 2 × 10^7^ TCMK-1 cells treated with or without 100 μM FeCl_2_ for 24 h with mitochondria isolated and subjected to mass spectrometry analysis. The PCA plot demonstrates distinct clustering between control and FeCl_2_-treated groups. (F) GSEA revealed significant upregulation of metal-binding proteins in the iron overload group, confirming successful model establishment. (G) Expression levels of [4Fe–4S]-containing proteins and assembly proteins. Protein levels were absolutely quantified and normalized to the NC group. Each cell represents the expression level of an individual protein in a single sample. (H) *In vitro* aconitase activity assay. Enzyme activity, which is closely associated with [4Fe–4S] cluster stability, was measured spectrophotometrically. Data are presented as mean ± SD, n = 3. (I) Western blot of mitochondria extracted from TCMK-1 cells post H/R 6 h, treated with FeCl_2_ (100 μM, 24 h) and control group. Data are presented as mean ± SD, n = 3 for all groups.

Mitochondrial proteomics revealed selective downregulation of [4Fe–4S] cluster-containing proteins and their assembly factors (ISCA2, NFU1, LIAS) in iron-overloaded tubular cells, with [2Fe–2S] systems remaining unaffected (Fig. 4G–S4B-C). Western blot confirmed reduced ISCA2, NFU1, and LIAS expression (Fig. 4I), paralleled by decreased aconitase activity under both iron overload and hypoxia-reoxygenation (Fig. 4H), representing [4Fe–4S] cluster destabilization. Notably, the [4Fe–4S] containing protein LIAS showed consistent depletion under both hypoxia-reoxygenation and iron-overload conditions, paralleling the observed deficiencies in iron-sulfur cluster assembly. The sustained downregulation of LIAS, a critical mediator of protein lipoylation necessary for copper-mediated TCA cycle interference, indicates that iron overload promotes cuproptosis by selectively impairing [4Fe–4S]-cluster-dependent pathways.

### 2.5 Iron overload results in the loss of LIAS iron-sulfur clusters and interferes with protein lipoylation

**Fig. 5:**
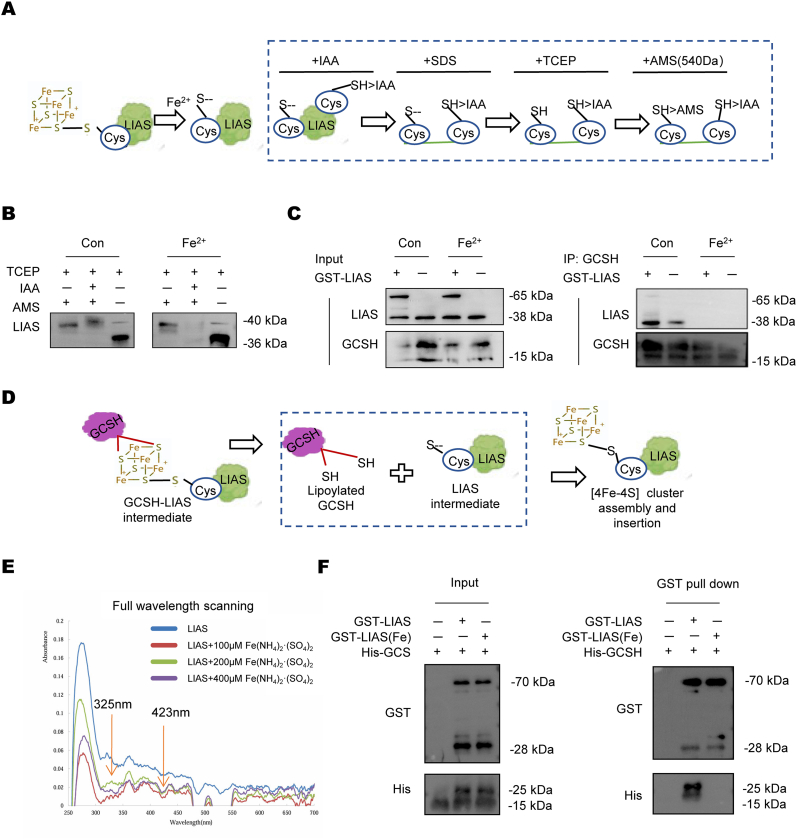
**Iron overload results in the loss of LIAS iron-sulfur clusters and interferes with protein lipoylation.** (A) Schematic representation of LIAS [4Fe–4S] cluster loss. In iron overload conditions, the [4Fe–4S] cluster is destabilized, exposing previously coordinated cysteine residues. (B) Schematic diagram of the IAA/AMS-based thiol trapping assay. (1) Membrane-permeable iodoacetamide (IAA) alkylates free thiols in intact cells. (2) Subsequent reduction converts disulfide-bonded cysteines to reactive thiols. (3) These thiols bind 540 Da AMS adducts, resulting in molecular weight shifts that reflect cysteine accessibility. (C) Western blot analysis of LIAS protein size changes using 10 % SDS-PAGE. The iron overload group exhibited varying degrees of cysteine residue exposure, indicating [4Fe–4S] cluster loss. (D) Co-IP analysis of interaction between LIAS and GCSH in FeCl_2_ (100 μM,24 h) treated or control TCMK-1 cells. Three biological repeated immunoblots have been performed. (E) Schematic representation of GCSH-LIAS interaction and iron-sulfur cluster transfer. Under physiological conditions, LIAS with an intact [4Fe–4S] cluster directly binds GCSH and facilitates iron-sulfur cluster transfer. Concurrently, the LIAS [4Fe–4S] cluster is replenished to ensure proper physiological function. In iron overload conditions, LIAS loses its [4Fe–4S] cluster, disrupting both GCSH binding and cluster transfer. (F) UV–visible absorption spectra of recombinant LIAS purified from the *E. coli* cells grown in LB medium supplemented with indicated concentration of (NH_4_)_2_Fe(SO_4_)_2_. The absorption peak at 325 nm and 423 nm indicates the binding of iron, and iron-sulfur cluster in proteins, respectively. (G) GST pull-down assay followed by Western blot analysis. Three biological repeated immunoblots have been performed.

To investigate iron overload-induced loss of LIAS [4Fe–4S] clusters, we employed an IAA/AMS-based reverse thiol trapping assay in normal and iron-overloaded cells. As shown in Fig. 5A, cluster dissociation in LIAS exposes its coordinating cysteines, enabling sequential modification: membrane-permeable iodoacetamide (IAA) first alkylates free thiols in intact cells, followed by reduction of disulfide-bonded cysteines to reactive thiols that bind 540 Da AMS adducts, with resultant molecular weight shifts reflecting cysteine accessibility (Fig. 5A). Reduced AMS labeling (lower molecular weight) indicates cluster retention, whereas increased AMS binding (higher molecular weight) signifies cluster loss. In LIAS, which coordinates two [4Fe–4S] clusters via seven cysteines, iron overload specifically led to the loss of iron-sulfur clusters and the exposure of cysteine residues (Fig. 5B). This finding demonstrates selective destabilization of the iron-sulfur clusters rather than nonspecific exposure of cysteines.

Co-immunoprecipitation assays first confirmed the endogenous interaction between LIAS and GCSH under physiological conditions (Fig. 5C). Importantly, this interaction was completely abolished in iron-overloaded cells, suggesting iron toxicity directly targets the LIAS-GCSH complex. Mechanistically, we demonstrated that LIAS mediates GCSH lipoylation through direct binding dependent on its [4Fe–4S] cluster (Fig. 5D). Thus, iron overload-induced Fe–S cluster degradation disrupts this essential protein-protein interaction, ultimately leading to lipoylation deficiency.

In prokaryotic systems, Fe (II) supplementation dose-dependently reduced the 423-nm [4Fe–4S] cluster UV absorption while enhancing free iron signals at 325 nm (Fig. 5E). GST pull-down assays further validated Fe–S cluster loss in Fe (II)-treated proteins, demonstrating iron overload-induced [4Fe–4S] destabilization across both eukaryotic and prokaryotic systems (Fig. 5F).

### 2.6 The loss of LIAS iron-sulfur clusters promotes cuproptosis

Previous studies have established that cuproptosis is driven by the pathological oligomerization of lipoylated proteins upon binding to toxic Cu (I) in mitochondria, leading to depletion of key lipoylated enzymes in the TCA cycle and subsequent cell death. Our prior findings demonstrated that iron overload induces LIAS [4Fe–4S] cluster loss, disrupting LIAS-mediated lipoylation and reducing lipoylated TCA cycle enzymes. Combined with Cu (I)-dependent pathological oligomerization of lipoylated proteins, these effects collectively impair TCA cycle function and trigger cell death, thereby sensitizing cells to copper-induced cytotoxicity.

**Fig. 6:**
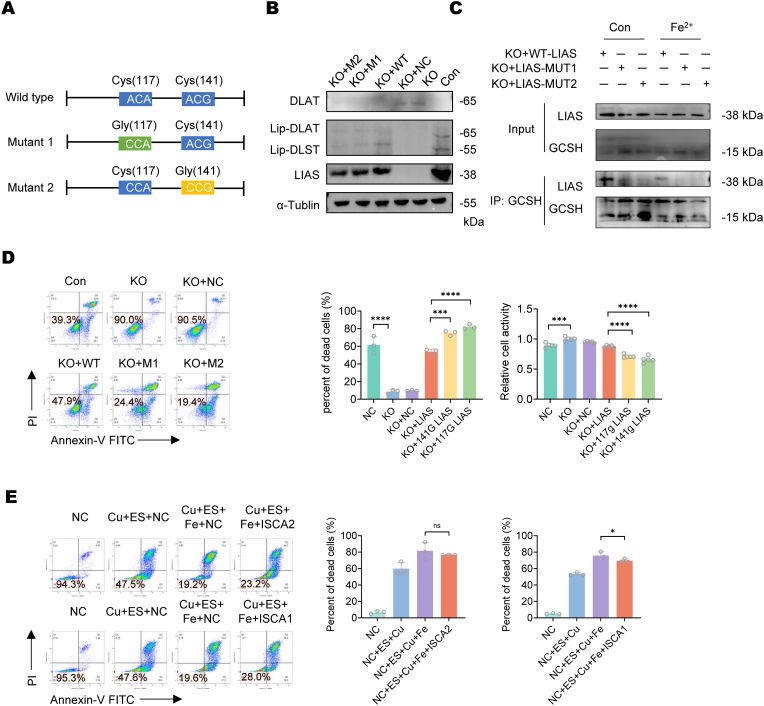
**The loss of LIAS iron-sulfur clusters promotes cuproptosis.** (A) Schematic representation of LIAS site-directed mutagenesis. Cysteine residues at positions 117 and 141, which coordinate the [4Fe–4S] cluster, were mutated to glycine (C117G and C141G). This mutation disrupts iron-sulfur cluster binding while maintaining protein structure. (B) Western blot analysis of LIAS knockout (KO) 293T cells transfected with LIAS point mutant overexpression plasmids. (C) Co-IP analysis of interaction between LIAS and GCSH in 293T cells transfected with or without LIAS point mutant overexpression plasmids or wt plasmids. (D) Cell death rate and cell viability analysis of LIAS KO 293T or control cells transfected with or without LIAS point mutant overexpression plasmids treated by elesclomol-Cu (II) (10 nM, 2 h). (E) Cell death analysis of TCMK-1 cells transfected with ISCA1 or ISCA2 overexpression plasmids treated by elesclomol-Cu (II) (10 nM, 2 h) with or without FeCl_2_ (100 μM, 24 h) treated. Dots represent individual data points. Data are presented as mean ± SD, n = 3 for all groups.

To investigate whether iron-sulfur cluster loss enhances copper-induced cell death, we generated a LIAS knockout (KO) 293T cell line and constructed plasmids with mutations at the iron-sulfur cluster coordination sites (Cys117 and Cys141) (Fig. 6A). In mutant LIAS-expressing cells, lipoylated protein levels were significantly reduced, confirming impaired lipoylation due to Fe–S cluster disruption (Fig. 6B). Co-immunoprecipitation assays further verified defective Fe–S cluster assembly in these mutants (Fig. 6C). When treated with equimolar ES-Cu, cells expressing mutant LIAS exhibited markedly higher mortality and increased sensitivity to copper-induced cell death compared to wild-type controls (Fig. 6D).

### 2.7 Overexpression of [4Fe–4S] assembly proteins partially rescues iron overload promoted cuproptosis

In Fig. 4, we showed that iron overload downregulates [4Fe–4S] assembly proteins, including ISCA1 and ISCA2, which mediate [2Fe–2S]-to-[4Fe–4S] cluster conversion [23]. To determine whether their reduced expression contributes to LIAS [4Fe–4S] cluster loss, we overexpressed ISCA1 and ISCA2 in iron-overloaded TCMK-1 cells and assessed cuproptosis sensitivity. Cells overexpressing ISCA1 exhibited reduced mortality upon FeCl_2_ and ES-Cu treatment and cells overexpressing ISCA2 showed a decreasing trend in mortality, though without reaching statistical significance. (Fig. 6E).

### 2.8 Iron chelation inhibits both cuproptosis and ferroptosis after RIRI

**Fig. 7:**
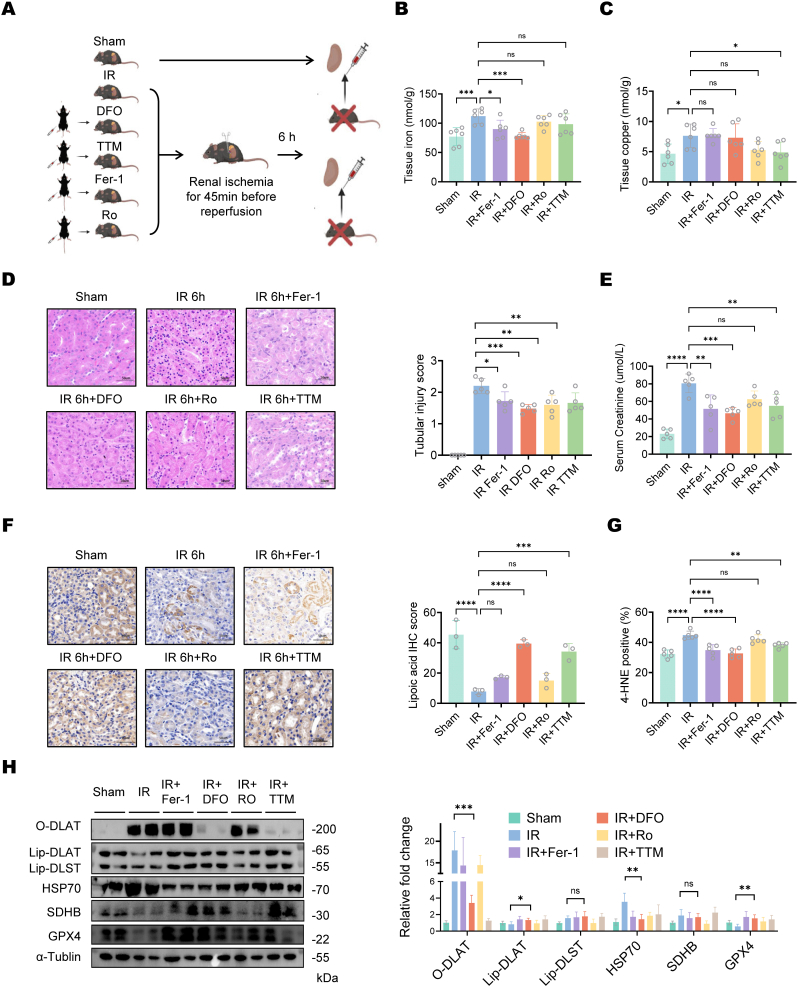
**Iron chelation most effectively inhibits both cuproptosis and ferroptosis after RIRI.** (A) Schematic representation of analysis of various treatments on renal ischemia-reperfusion injury (IRI). Mice were subjected to 45 min of renal ischemia followed by reperfusion. Treatments included deferoxamine (DFO, 100 mg/kg), tetrathiomolybdate (TTM, 10 mg/kg), ferrostatin-1 (Fer-1, 5 mg/kg), and rosiglitazone (Ro, 5 mg/kg). Each compound was delivered 5, 3, and 1 days prior to model induction Samples were collected 6 h post-reperfusion. Sham-operated mice served as controls. (B) Tissue iron concentration of mice in different group. Data are presented as mean ± SD, n = 5. (C) Tissue copper concentration of mice in different group. Data are presented as mean ± SD, n = 5. (D) Representative H&E staining of renal tissues from different treatment groups. Tubular injury score of mice in different group were shown in bar chart. Data are presented as mean ± SD, n = 5.(Magnification: × 200, Scale bar: 50 μm) (E) Serum creatinine of mice in different group. Data are presented as mean ± SD, n = 5. (F) Lipoic acid IHC staining of mice kidney tissues indicated significantly increased lipoylation levels in the DFO group compared to IR controls (Magnification: × 200, Scale bar: 50 μm). Quantification of Lipoic acid IHC staining score across different treatment groups were shown in bar chart. Data are presented as mean ± SD, n = 5. (G) Quantification of positive area of 4-HNE IHC staining across different groups. Data are presented as mean ± SD, n = 5. (H) Western blot of mice kidney tissue post RIRI in different group. Data are presented as mean ± SD, n = 5.

**Fig. 8:**
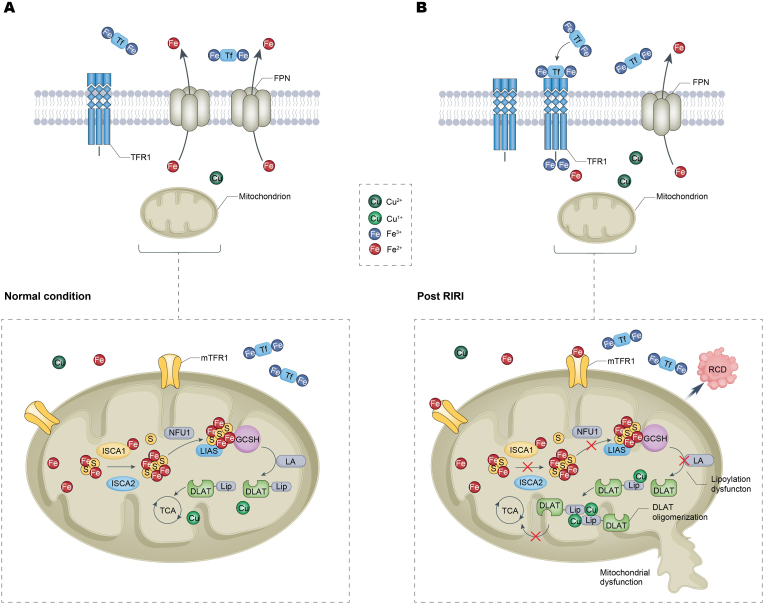
**Mechanism hypothesis diagram.** Under normal conditions, transferrin receptor 1 (TFR1) on the cell membrane facilitates the uptake of iron from circulating iron ions and iron-bound transferrin into the cell, where it is reduced to ferrous iron (Fe^2+^). Mitochondrial TFR1 (mTFR1) then transports Fe^2+^ into the mitochondria, where it participates in the synthesis of [2Fe–2S] clusters, which are further assembled into [4Fe–4S] clusters with the mediation of ISCA1, ISCA2, and NFU1. These [4Fe–4S] clusters are inserted into the LIAS protein, enabling its interaction with GCSH and facilitating the lipoylation of mitochondrial proteins such as DLAT and DLST, which are essential for the TCA cycle and normal cellular respiration. In contrast, under ischemia-reperfusion conditions, the upregulation of TFR1 and mTFR1 increases iron uptake, while the downregulation of ferroportin (FPN) reduces iron efflux, leading to elevated intracellular and mitochondrial Fe^2+^ levels. Concurrently, the decreased expression of ISCA1, ISCA2, and NFU1 disrupts [4Fe–4S] cluster assembly and stability, impairing LIAS-GCSH interaction and lipoylation. This results in incomplete lipoylation of DLAT and other proteins, some of which form toxic oligomers by binding to mitochondrial copper ions. Both non-lipoylated and oligomerized proteins fail to participate in the TCA cycle, ultimately leading to cell death.

Based on previous findings, we further validated *in vivo* that iron chelators alleviate RIRI by dual inhibition of cuproptosis and ferroptosis, using other ferroptosis inhibitors and cuproptosis inhibitors as controls (Fig. 7A). We assessed renal injury status and tubular cell cuproptosis and ferroptosis marker in each group 6 h post-operation. The renal tissue analysis revealed that both DFO and Fer-1 treatments significantly reduced Fe (II) levels compared to untreated controls, while other treatments showed no such effect (Fig. 7B). In contrast, copper ion concentrations were only decreased in the group receiving copper-specific chelators (Fig. 7C). Results showed that mice treated with all inhibitors of ferroptosis and cuproptosis exhibit significantly lower levels of serum creatinine and tissue damage scores compared to the control group (Fig. 7D and E). These findings further confirmed that cell cuproptosis and ferroptosis can occur during RIRI. Moreover, we found both Fer-1 and the iron chelator DFO could alleviate tubular cell cuproptosis, as demonstrated by elevated lipoic acid levels compared to controls (Fig. 7F), along with decreased oligomerized DLAT and HSP70 expression and increased Fe–S cluster protein SDHB levels (Fig. 7H). In contrast, Ro treatment showed no significant effects on these molecular markers. These differential responses confirm that Fer-1 and DFO, but not Ro, effectively mitigate cuproptosis in tubular cells (Fig. 7G and H). Copper chelator TTM can also partially inhibit cell ferroptosis (Fig. 7G–S2E). Our results demonstrate that iron chelation represents the most effective strategy for simultaneously inhibiting both cuproptosis and ferroptosis, thereby providing a novel therapeutic approach for preventing renal ischemia-reperfusion injury (see Fig. 8).

## 3 Discussion

Metal ion homeostasis is a tightly regulated process that becomes disrupted in disease states [24,25]. Numerous studies have documented altered metal ion concentrations in various pathologies, with recent attention focusing on metal overload-induced cell death [26,27]. Yogesh Scindia et. raised iron overload post RIRI and some studies have reported disruption of metal ion homeostasis plays a crucial role in the development of RIRI [28]. In our study, we focused on the coordinated changes in copper and iron ions. Serum analysis revealed an initial decrease followed by iron rebound, contrasting with copper trends. Since changes in blood do not necessarily represent local tissue condition, we examined the variations in copper and iron ions in renal tissue. Tissue-level analysis showed iron accumulation without corresponding copper increases, suggesting compartment-specific regulation. Mechanistically, TFR1 upregulation and FPN downregulation drove tissue iron retention, while CTR1 downregulation limited copper uptake. Notably, cuproptosis and ferroptosis markers emerged within 6 h post-reperfusion, preceding overt copper overload. Given that both pathways are triggered by metal ion overload, we hypothesized potential cross-regulation between cuproptosis and ferroptosis, particularly in the context of tissue copper accumulation and copper-induced cell death.

Previous studies have shown that copper overload can exacerbate oxidative stress through the Fenton reaction, while also promoting iron overload [12,29]. Additionally, copper overload has been found to facilitate the ubiquitination and degradation of GPX4, thereby promoting ferroptosis. We cross-examined the renal injury following the application of inhibitors for cuproptosis and ferroptosis [30,31]. The experimental results indicated that ferroptosis inhibitors could partially alleviate copper-induced cell death, with iron chelators showing particularly stable and significant effects. Therefore, we hypothesize that iron overload may play a role in promoting copper-induced cell death. And the results confirmed this hypothesis.

In iron-overloaded cells, we observed mitochondrial downregulation of both [4Fe–4S]-containing proteins and their assembly machinery. Iron-sulfur clusters are known to play a central role in regulating intracellular iron homeostasis through modulation of iron regulatory proteins (IRPs). [32,33]. Under iron-deficient conditions, reduced Iron-sulfur clusters synthesis leads to increased IRP activity, which in turn promotes Tfr1 expression, suppresses ferritin translation, and decreases FPN expression, thereby facilitating iron uptake and reducing export to restore iron availability. [32]. Under normal conditions, Fe–S cluster biosynthesis also consumes free iron, contributing to iron buffering. However, the Fe–S cluster defects observed in this study under conditions of iron overload may not be fully explained by altered IRP signaling alone. These findings suggest the possibility of a distinct mitochondrial response to excess iron.

Cuproptosis is driven by mitochondrial Cu (I) induced oligomerization of lipoylated proteins, leading to depletion of lipoylated TCA cycle enzymes and subsequent cell death [20]. Central to this process is LIAS, a [4Fe–4S] cluster-dependent lipoic acid synthase that catalyzes the essential lipoylation modification required for TCA cycle. Our findings demonstrate that iron overload disrupts this pathway through ISCA1 and ISCA2 downregulation, key [4Fe–4S] assembly proteins, resulting in LIAS [4Fe–4S] cluster loss and impaired lipoylation function. These defects generate aberrantly lipoylated proteins that potentiate Cu (I) dependent oligomerization, while also reducing the availability of properly lipoylated proteins for participation in the TCA cycle. Both effects exacerbate TCA cycle dysfunction and cell death. Notably, ISCA1 re-expression partially rescues cuproptosis, supporting its role in maintaining LIAS function. Thus, iron overload likely sensitizes cells to cuproptosis through ISCA1/2-mediated LIAS dysfunction, although the precise relationship between ISCA1/2 downregulation and iron overload requires further investigation.

In summary, our study demonstrates that renal ischemia-reperfusion injury disrupts copper-iron homeostasis, triggering a cascade of tubular epithelial cell damage. Iron overload sensitizes cells to cuproptosis through downregulation of [4Fe–4S] assembly proteins (particularly ISCA1/2), leading to LIAS iron-sulfur cluster deficiency, defective lipoylation, and ultimately copper-induced cell death. These findings not only elucidate a novel mechanism of ischemia reperfusion injury but also highlight the therapeutic potential of iron chelation.

However, systemic iron chelation may not be ideal for end-stage renal disease (ESRD) patients, who often require oral iron supplementation for anemia management [34]. Moreover, prolonged or excessive iron chelation may paradoxically exacerbate mitochondrial iron accumulation through iron regulatory protein (IRP)-mediated feedback [35]. Instead, the targeted application of iron chelators in organ preservation and perfusion solutions offers a promising alternative [36]. This approach can mitigate graft iron overload during transplantation, potentially reducing DGF incidence and improving transplant outcomes. Additionally, using iron chelators in preservation solutions avoids the need for prolonged systemic administration, thereby minimizing the risk of IRP pathway activation and its associated adverse effects. This organ-specific strategy warrants further investigation as a means to protect against ischemia-reperfusion injury while preserving systemic iron homeostasis.

## 4 Materials and methods

### 4.1 Study design and data source

This retrospective study was conducted using the clinical database of Zhongshan Hospital, Fudan University from September 2023 to June 2024. Patients who underwent kidney transplantation with complete data were included in the study. Cases were defined as patients with histologically confirmed delayed graft function (DGF) (n = 9) according to AST Consensus Conference (2021), while controls (n = 9) were randomly selected from individuals without DGF. The study was approved by the Ethics Committee of Zhongshan Hospital, Fudan University (No. B2024-303), with waived informed consent for anonymized retrospective data.

### 4.2 Animal models

Male C57BL/6 mice (6–8 weeks old) were obtained from SLAC Laboratory Animal Co., Ltd. (Shanghai, China). All animal experiments were approved by the Animal Welfare & Ethics Committee of Zhongshan Hospital and in compliance with ethical guidelines and procedures (2024-215). To establish an ischemia-reperfusion injury model, unilateral renal pedicle clamping was performed for 45 min on one kidney, while the contralateral kidney underwent ligation and excision of the renal pedicle. During surgery, mice were maintained on a thermostatic pad (37 °C) to preserve normothermia. After 45 min of ischemia, clamps were removed to initiate reperfusion. Sham-operated mice underwent identical procedures without renal pedicle clamping. Groups of animals were euthanized under isoflurane anesthesia at predefined time points (3, 6-, 12-, 24-, and 48-h post-ischemia) for downstream analyses.

To investigate the preventive effects of various ferroptosis inhibitors and cuproptosis inhibitors on renal ischemia-reperfusion injury, as well as their protective roles against cuproptosis and ferroptosis, the following pharmacological agents were administered via intraperitoneal injection to respective experimental groups at 5, 3, and 1 days prior to model induction: ferrostatin-1 (Fer-1, 5 mg/kg), deferoxamine (DFO, 100 mg/kg), rosiglitazone (RO, 5 mg/kg), and ammonium tetrathiomolybdate (TTM, 10 mg/kg). Each compound was delivered according to established dosing protocols through standardized intraperitoneal injection procedures to ensure precise temporal administration relative to disease model establishment. The control group subjects received intraperitoneal injections of 0.1 mL vehicle control (solvent) at matching time points (5, 3, and 1 days prior to model induction) to maintain parity in procedural variables.

Elesclomol and copper sulfate (CuSO_4_) were mixed at equimolar concentrations to form the Cu-Elesclomol complex (ES-Cu) dissolved in a solvent containing 10 % DMSO, 40 % PEG300, 5 % Tween-80, and 45 % saline. Mice received an intraperitoneal injection of 0.1 mL Cu-Elesclomol complex (100 μmol/kg body weight, calculated based on Elesclomol concentration). The intervention group was treated with an intraperitoneal injection of 100 mg/kg DFO in combination with ES-Cu.

To establish a mouse model with local iron overload in renal tubular epithelial cells. Six-week-old male mice received 0.2 mL of AAV9-Ksp-GFP-shFPN (2.0 × 10^8^ pfu/ml) or control vector via tail vein injection. Model induction was performed 30 days post-injection.

### 4.3 Cell culture

Mouse renal tubular epithelial cells (TCMK-1 cell line) from ATCC were cultured in HyClone Dulbecco's modified Eagle's medium (DMEM)/F12 supplemented with 5 % fetal bovine serum (FBS) under 5 % CO_2_ conditions. Human embryonic kidney 293T (HEK293T) cells were maintained in high-glucose DMEM supplemented with 10 % FBS and 1 % penicillin-streptomycin. For hypoxia-reoxygenation treatment, cells were exposed to conditions (5 % CO_2_, 1 % O_2_, and 94 % N_2_) for 24 h, followed by reoxygenation for either 2, 4 or 6 h.

### 4.4 Serum creatinine measurement in mice

Blood samples were collected from mice via retro-orbital puncture under anesthesia and centrifuged at 3000×*g* for 10 min at 4 °C to isolate serum. The Quantichrom™ Creatinine Assay Kit (DICT-500, BioAssay Systems, Hayward, CA) was used for quantitative determination of creatinine levels. All assays were performed following manufacturer's instructions.

### 4.5 Serum and kidney tissue iron measurement

For mice kidney tissue Iron Measurement. Kidney tissues (100 mg) were weighed, rinsed with cold PBS, homogenized in 10 vol of Assay Buffer, sonicated for 5 min, and centrifuged at 16,000×*g* for 10 min to collect supernatants; serum samples were diluted 1:2 with Assay Buffer to ensure iron concentrations within the standard curve range. A 100 μM Fe^2+^ standard solution was prepared by diluting the 1 mM stock with Assay Buffer, followed by serial 2-fold dilutions (0–50 μM), with 5 % Reducer solution (total iron) or Assay Buffer (Fe^2+^) added to standards. For detection, 400 μL of kidney supernatants or diluted serum were aliquoted into three tubes: Fe^2+^ samples (5 % Assay Buffer), total iron samples (5 % pre-dissolved Reducer solution), and sample blanks (5 % Assay Buffer); all tubes were incubated at 37 °C for 15 min. Subsequently, 105 μL of standards, samples, and blanks were transferred to a 96-well plate (triplicates), mixed with 100 μL Probe Solution (standards and samples) or Assay Buffer (blanks), incubated at 37 °C for 1 h, and absorbance was measured at 593 nm. Fe^2+^ and total iron concentrations were calculated using the standard curve (absorbance vs. Fe^2+^), with Fe^3+^ derived by subtracting Fe^2+^ from total iron; dilution factors were applied to report original sample concentrations.

### 4.6 Serum and kidney tissue copper measurement

Kidney tissues were homogenized in distilled water, centrifuged to collect supernatants, and protein concentrations were determined using a BCA kit; serum samples were used undiluted. A copper standard series (0–60 μmol/L) was prepared by diluting the 100 μmol/L stock with distilled water. Chromogenic working solution (Reagent 1: Reagent 2 = 14:1) was freshly mixed. For detection, 15 μL of standards or samples were added to a 96-well plate, followed by 230 μL chromogenic working solution. After incubation at 37 °C for 5 min, absorbance was measured at 580 nm. Tissue copper concentrations (μmol/g) were calculated using the standard curve and normalized to protein content; serum copper levels (μmol/L) were derived directly from the curve. All samples were analyzed in triplicate.

### 4.7 Western blotting

Freshly isolated mouse kidney tissues or cells or extracted mitochondria were lysed in RIPA buffer (Yeasen, China) supplemented with protease inhibitors. After centrifugation, protein concentrations in supernatants were quantified using a BCA assay kit (Beyotime, China). Proteins (50 μg per lane) were separated by SDS-PAGE (10 % or 12.5 % gels) and transferred to PVDF membranes. Membranes were blocked with 5 % non-fat milk and incubated overnight at 4 °C with primary antibodies against: DLAT (Proteintech, 68303-1-Ig, 1:5000), HSP70 (Proteintech, 10995-1-AP, 1:5000), SDHB (Proteintech, 10620-1-AP, 1:5000), GCSH (Proteintech, 16726-1-AP, 1:500), SLC40A1 (Proteintech, 26601-1-AP, 1:100), α-Tublin (Proteintech, 14555-1-AP, 1:5000), ISCA2 (Proteintech, 13200-1-AP, 1:1000), ATP5A1 (Proteintech, 66037-1-Ig, 1:5000), COX-1 (Proteintech, 13393-1-AP, 1:500), NFU1 (Proteintech, 25598-1-AP, 1:500), 6∗His (Proteintech, 66005-1-Ig, 1:5000), GST (Proteintech, 66001-2-Ig, 1:5000), LIAS (Proteintech, 11577-1-AP, 1:1000), GPX4 (Abcam, ab252833, 1:1000), Anti-Lipoic Acid Rabbit pAb (Millipore, 437695, 1:1000). After washing, membranes were incubated with HRP-conjugated secondary antibodies for 1 h at room temperature. Protein bands were visualized using ECL reagent and analyzed with ImageJ (v1.8.0).

### 4.8 Histological, immunohistochemical and immunofluorescent analysis

Kidney sections from different mouse groups were fixed in 4 % paraformaldehyde (PFA) and processed through standard histological procedures: fixation, dehydration, paraffin embedding, and sectioning. Hematoxylin and eosin (H&E) staining was performed for histological analysis using light microscopy. Tubular injury severity was scored on a scale of 0–5 based on cellular necrosis and brush border loss: 0 (none), 1 (0–10 %), 2 (11–25 %), 3 (26–45 %), 4 (46–75 %), and 5 (>75 %). For immunohistochemistry, sections were incubated overnight at 4 °C with primary antibodies against 4-HNE (Abcam, ab48506, 1:25), lipoic acid (Millipore, 437695, 1:600), and SLC40A1 (Proteintech, 26601-1-AP, 1:100), followed by 1 h incubation with secondary antibodies at room temperature. After DAB staining and hematoxylin counterstaining, slides were imaged using an Olympus IX83 microscope. For immunofluorescent analysis, kidney tissues or cell samples were fixed in 4 % formalin (2 h, room temperature), dehydrated in 30 % sucrose overnight, and embedded in paraffin. Cryosections (5–10 μm) were blocked with 10 % donkey serum. Primary antibodies against DLAT (Proteintech, 68303-1-Ig, 1:100), GPX4 (Abcam, ab314333, 1:100) were incubated for 1 h, followed by PBS washes. Secondary antibodies (Cy3, AF488, HRP; Servicebio, 1:300–1:400) were applied for 45 min. Nuclei were stained with DAPI (1:10,000, Cell Signaling), and slides were mounted with ProLong Gold (Invitrogen). Fluorescence signals were captured using appropriate excitation/emission wavelengths.

### 4.9 RNA extraction and qPCR analysis

Total RNA was isolated from mouse kidney tissues and TCMK-1 cells using TRIzol reagent (Sigma). Reverse transcription was performed with the PrimeScript RT Kit (Takara). Quantitative PCR (qPCR) was carried out using SYBR Green Master Mix (Yeasen) on an ABI Prism 7900HT system (Applied Biosystems). Gene expression levels were normalized to β-actin and calculated via the 2−ΔΔCt method. Primer sequences are listed in the Supplementary Table.

### 4.10 Living cell mitochondria and Fe^2+^ co-localization staining and imaging

Staining for mitochondrial and Fe^2+^ co-localization was performed using MitoTracker® Green CMTMRos (Invitrogen, M7510) and FerroOrange (Dojindo, F374) following the manufacturer's protocols. Cells were seeded onto glass-bottom dishes and cultured overnight at 37 °C with 5 % CO_2_. For staining, a working solution was prepared by diluting 100 nM MitoTracker® Green CMTMRos and 1 μM FerroOrange in 1 mL of HBSS. Cells were incubated with the solution for 30 min at 37 °C in the dark, followed by three washes with HBSS.

Mitochondrial membrane potential was assessed using tetramethylrhodamine methyl ester (TMRM) (Thermo Fisher Scientific, I34361) in combination with MitoTracker® Green CMTMRos (Invitrogen, M7510). Cells were seeded onto glass-bottom dishes and cultured overnight at 37 °C with 5 % CO_2_. For staining, a working solution of 100 nM MitoTracker® Orange CMTMRos and 100 nM TMRM was prepared in pre-warmed serum-free medium. Cells were incubated with the staining solution for 30 min at 37 °C in the dark, followed by three washes with HBSS to remove excess dye.

After staining, live cells were immediately imaged without fixation using an FV300 confocal laser scanning microscope (Olympus) with excitation/emission settings of 488/519 nm for MitoTracker® Green CMTMRos and 561/610 nm for FerroOrange. Fluorescence co-localization analysis was performed using ImageJ, with fluorescence intensity profiles generated to assess signal overlap.

### 4.11 Cell death assay

Cell death was assessed using Annexin V-FITC and propidium iodide (PI) staining. Cells were plated at a density of 2 × 10^5^ cells per well in 6-well plates and allowed to adhere overnight. The following day, cells were treated as indicated. For Annexin V-FITC-PI staining, cells were harvested, washed with binding buffer, and then incubated in binding buffer containing ANXA5-FITC and PI (Yeasen, 40302ES60) for 15 min in the dark. Flow cytometry was used to quantify dead cells.

### 4.12 Cell viability assay

Cell viability was assessed using the Cell Counting Kit-8 (CCK-8; Yeasen, 40203ES76). Cells were seeded at a density of 1 × 10^4^ cells per well in 96-well plates and treated with the indicated drugs. After treatment, 100 μL of fresh medium containing 10 μL of CCK-8 solution was added to each well, followed by incubation for 1 h at 37 °C in a 5 % CO_2_ incubator. The absorbance at 450 nm was then measured using a microplate reader (Thermo Scientific, Varioskan Flash).

### 4.13 Lipid peroxidation assay

Lipid peroxidation was evaluated using BODIPY 581/591C11 staining (Dojindo, L267). Cells were seeded at a density of 1 × 10^5^ cells per well in 12-well plates and treated as indicated under 5 % CO_2_ at 37 °C. In the final 30 min of incubation, cells were exposed to 1.5 μM BODIPY 581/591C11 dye. After staining, cells were washed twice with HBSS, resuspended in HBSS, and analyzed by flow cytometry.

### 4.14 ICP-MS

For cell pellet samples, digestion was performed using 0.2 mL of nitric acid (HNO_3_) and 0.05 mL of hydrogen peroxide (H_2_O_2_) in a microwave digestion system at 120 °C for 30 min, followed by dilution with 0.25 mL of pure water. All processed samples were spiked with 50 μL of indium (In) (100 ng/mL) as an internal standard and thoroughly mixed by vortexing before analysis. Multi-element standard solutions containing Cu, Fe, and a working internal solution of In were prepared from individual element stock solutions obtained from LGC. Elemental analysis was performed using an Agilent 7900 ICP-MS system, with Cu, Fe, and In detected at mass-to-charge ratios of 63, 56 respectively.

### 4.15 Detection of aconitase activities

The aconitase (ACO) activity assay was performed using the Boxbio Aconitase Activity Assay Kit (AKAC008 M) following the manufacturer's protocol. Cell pellets (5 × 10^6^–10^7^ cells) were homogenized in Reagent 1 (lysis buffer) supplemented with Reagent 3 (volatile component) and subjected to ice-bath sonication (300 W, 3 s on/9 s off, 15 cycles). For total ACO extraction, homogenates were centrifuged at 11,000 g (4 °C, 15 min), and supernatants were diluted 5–10 × with distilled water prior to assay. To isolate cytosolic and mitochondrial fractions, homogenates were first centrifuged at 600 g (4 °C, 5 min) to pellet debris, followed by 11,000 g (4 °C, 15 min) to separate cytosolic (supernatant) and mitochondrial (pellet) fractions. Mitochondrial pellets were resuspended in Reagent 2 (extraction buffer) with Reagent 3, sonicated, and centrifuged at 5000 g (2 min) to collect mitochondrial extracts. For activity measurement, pre-warmed Reagent 4 (180 μL) was combined with 20 μL diluted sample (total, cytosolic, or mitochondrial extract) in a 96-well UV plate. Absorbance at 240 nm was recorded immediately (A_1_ at 10 s) and after 300 s incubation at 25 °C (A_2_ at 310 s). ACO activity was calculated using ΔA (A_2_ – A_1_).

### 4.16 Proteomic data processing and statistical analysis

Mitochondrial isolation was performed using the Thermo Scientific™ Mitochondria Isolation Kit for Cultured Cells (Product No. 89874) according to the manufacturer's instructions. The sample was transferred it to a 1.5 ml centrifuge tube and lysed with DB lysis buffer (8 M Urea, 100 mM TEAB, pH 8.5), followed by 5 min of ultrasonication on ice. The lysate was centrifuged at 12,000 g for 15 min at 4 °C and the supernatant was added with 1 M DTT to react for 1 h at 56 °C, and subsequently alkylated with sufficient iodoacetamide for 1 h at room temperature in the dark followed by ice-bath for 2 min. After quality test, each protein sample was taken and the volume was made up to 100 μL with DB lysis buffer (8 M Urea, 100 mM TEAB, pH 8.5), trypsin and 100 mM TEAB buffer were added, sample was mixed and digested at 37 °C for 4 h. Then trypsin and CaCl_2_ were added digested overnight. Formic acid was mixed with digested sample, adjusted pH under 3, and centrifuged at 12,000 g for 5 min at room temperature. The supernatant was slowly loaded to the C18 desalting column, washed with washing buffer (0.1 % formic acid, 3 % acetonitrile) 3 times, then added elution buffer (0.1 % formic acid, 70 % acetonitrile). The eluents of each sample were collected and lyophilized.

Prepare mobile phase A (100 % water, 0.1 % formic acid) and B (80 % acetonitrile, 0.1 % formic acid). The lyophilized powder was dissolved using 10 μ LA solution, centrifuged at 14,000g for 20 min at 4 °C, and 200 ng of the supernatant sample was injected into the sample for liquid-quality detection. A Vanquish Neo upgraded UHPLC system was used with a C18 pre-column of 174500 (5 mm × 300 μm,5 μm, Thermo) heated at 50 °C in a column oven, and a C18 analytical column of ES906 (PepMap TM Neo UHPLC 150 μm × 15 cm, 2 μm, Thermo), and the elution conditions of the liquid chromatography were as shown in Table 1, a Thermo orbitrap astral mass spectrometer mass spectrometer was used, an Easy-spray (ESI) ion source was used, the ion spray voltage was set to 1.9 kV, the ion transfer tube temperature was set to 290 °C, and the mass spectrum was in a data dependent acquisition mode, with a full first-stage mass spectrometry scanning range of *m/z* 380–980. The primary MS resolution was set to 240,000 (200 *m/z*), AGC was set to 500 %, the parent ion window size was set to 2-Th, the number of DIA windows was 300, the NCE was set to 25 %, the secondary *m/z* acquisition range was from 150 to 2000, the sub-ion resolution Astral was set to 80,000, and the maximal injection time was 3 ms. Into mass spectrometry detection raw data.

**Table 1:** Baseline characteristics for DGF and non-DGF patients.

Variables	Non-DGF (n = 9)	DGF (n = 9)	P value
Age, mean(±SD)	33.25 ± 6.017	39.00 ± 11.22	0.1404
Female (%)	44.4 %	22.2 %	
Cold ischemia time (h, mean ± SD)	3.611 ± 3.516	5.556 ± 1.878	0.1627
Warm ischemia time (min, mean ± SD)	28.33 ± 6.124	33.33 ± 6.614	0.1156
Recipient preoperative hemoglobin (g/dL, 95 % CI)	101.8 (85.66,117.9)	100.4 (85.08,115.8)	0.8919
Recipient preoperative serum creatinine (μmol/L, 95 % CI))	785.3 (602.3968.3)	869.1 (662.4,1076)	0.4957
Recipient preoperative BUN (mg/dL, 95 % CI))	24.43 (19.48,29.39)	20.79 (17.43,24.15)	0.1794
Recipient preoperative total iron-binding capacity (95 % CI)	46.78 (34.85,58.7)	51.44 (42.82,60.05)	0.4761
Recipient preoperative transferrin saturation (95 %CI)	26.33 (17.97,34.70)	20.67 (15.63,25.71)	0.2523
Recipient preoperative serum iron (μmol/L, 95 % CI)	11.07 (6.95,15.18)	9.51 (6.814,12.21)	0.4700
Recipient preoperative serum copper (μmol/L, 95 % CI)	12.79 (6.741,18.84)	10.19 (5.689,14.69)	0.4380

The normal range for serum iron is 11–30 μmol/L and for serum copper is 11–24.4 μmol/L.

The raw files were searched and analyzed using the DIA-NN library search software. The library search parameters were set as follows: a mass tolerance of 10 ppm for precursor ions and 0.02 Da for fragment ions. Cysteine was modified by alkylation, methionine was oxidatively modified, and N-terminal modifications included acetylation, loss of methionine, and loss of methionine + acetylation. One missed cleavage site was allowed at most.

To improve the quality of the analytical results, the DIA-NN software further filtered the search results by retaining only credible Peptide Spectrum Matches (PSMs) with a confidence level of 99 % protein or higher. Only credible spectral peptides and proteins were retained, and FDR validation was performed to remove peptides and proteins with an FDR greater than 1 %. A protein with a fold change (FC) greater than or less than a certain value (FC) was defined as a differentially expressed protein (DEP).

### 4.17 Coimmunoprecipitation (Co-IP)

The experiment was conducted following the protocol provided by MedChemExpress (Monmouth Junction, NJ, USA). Cell lysates were collected and subjected to immunoprecipitation using either an anti-GCSH antibody at 4 °C for 4 h. Subsequently, 20 μl of protein A/G magnetic beads (MedChemExpress) were added to the lysates, and the mixture was rotated at 4 °C overnight. Following the overnight incubation, the beads were washed three times with immunoprecipitation buffer. The immunoprecipitates were then eluted by boiling in 1 % (wt:vol) SDS sample buffer at 100 °C for 5 min, after which they were analyzed by western blotting. Equal amounts of the extracts were separated by SDS-PAGE, transferred onto nitrocellulose membranes, and probed with specific antibodies.

### 4.18 Protein purification under varied conditions

The protein purification was conducted by BIORTUS using the recombinant construct pET28a-StrepII-GST-TEV-GG-LIAS (L28-L372) expressed in *E. coli* BL21 (DE3) cells under three distinct conditions to evaluate iron source effects. Cells were cultured in LB medium supplemented with 0.5 mM IPTG at 15 °C for 16 h, with variations including LB alone (baseline), LB + 100 μM FeCl_2_, LB + 100 μM Fe(NH_4_)_2_(SO_4_)_2_, LB + 200 μM Fe(NH_4_)_2_(SO_4_)_2_, LB + 400 μM Fe(NH_4_)_2_(SO_4_)_2_. Following lysis in a buffer containing 50 mM Tris-HCl (pH 8.0), 500 mM NaCl, and 5 % glycerol, lysates were clarified by centrifugation to isolate supernatant, which was then incubated with 50 μL Strep-Tactin XT resin for affinity purification. After washing with the same buffer, bound protein was eluted using 75 mM biotin, and the eluate (enriched in StrepII-GST-TEV-GG-LIAS) was treated with TEV protease to cleave the StrepII tag and linker sequences, yielding the final product GST-LIAS. Purification efficiency was confirmed by SDS-PAGE (4–20 % gradient gel) showing a molecular weight of ∼66.6 kDa (theoretical MW: 66599.27 Da), with Nanodrop A280 quantification and anti-GST Western blotting validating purity. All conditions demonstrated effective target enrichment via Strep-Tactin XT resin, and residual tags/protease were removed by size-exclusion chromatography.

### 4.19 GST-pull down assay

The GST pull-down assay was performed using BeyoGold™ GST-tag Purification Resin to investigate protein-protein interactions. Briefly, 50 μL of 50 % resin slurry was equilibrated with 20 × volume of GST pull-down protein binding buffer, centrifuged at 1000×*g* for 2 min, and resuspended in an equal volume of binding buffer. For the binding reaction, 400 μL of binding buffer, 50 μL of equilibrated resin, 25 μg of target protein (6∗HIS-GCSH), and either 25 μg of GST-tagged bait protein (GST-LIAS, GST-LIAS[Fe]) was combined in separate tubes, adjusted to a final volume of 0.5–1 mL, and incubated on a rotary shaker at 4 °C for 2 h or overnight. After incubation, the resin was centrifuged at 1000×*g* for 2 min, and the supernatant was collected for optional analysis. The resin was washed three times with 100 μL of GST pull-down wash buffer, followed by elution with 50 μL of elution buffer (10 min incubation) or direct boiling in 25 μL of 2 × SDS-PAGE loading buffer (5 min, 95 °C). Eluted proteins were analyzed by SDS-PAGE and detected via Western blotting.

### 4.20 Reverse thiol trapping assay

Pretreated cells were resuspended in 80 mM cell-permeable iodoacetamide (IAA) to alkylate free thiol groups, followed by a 30-min incubation at room temperature. The samples were then washed twice with PBS and resuspended in 30 μL PBS. To reduce oxidized cysteine residues, 5 mM tris (2-carboxyethyl) phosphine (TCEP) and 2 % SDS were added, and the mixture was heated at 95 °C for 12 min, then cooled to room temperature. Subsequently, 4.5 mM 4-acetamido-4′-maleimidylstilbene-2,2′-disulfonic acid (AMS) was introduced to block newly exposed free cysteines. Finally, samples were mixed with Laemmli sample buffer and subjected to 10 % SDS-PAGE, followed by immunoblotting using target-specific antibodies.

### 4.21 Statistical analysis

Data are presented as the mean ± SD from at least three independent biological replicates. For comparisons between two groups, statistical significance was determined using Student's t-test, assuming normal distribution of the data. For comparisons involving three or more groups, one-way ANOVA followed by appropriate post-hoc multiple comparison tests (Tukey's or Dunnett's test) was applied. A P-value <0.05 was considered statistically significant. All statistical analyses were conducted using GraphPad Prism 8 software.

## CRediT authorship contribution statement

**Siyue Chen:** Writing – original draft, Visualization, Validation, Software, Methodology, Investigation, Formal analysis, Data curation, Conceptualization. **Tingting Chen:** Writing – review & editing, Investigation, Formal analysis, Conceptualization. **Cuidi Xu:** Visualization, Validation, Software, Methodology, Formal analysis. **Xiaohan Yu:** Methodology, Investigation, Formal analysis. **Junyu Shi:** Methodology, Investigation. **Cheng Yang:** Writing – review & editing, Visualization, Validation, Supervision, Resources, Project administration, Funding acquisition, Data curation, Conceptualization. **Tongyu Zhu:** Writing – review & editing, Supervision, Resources, Project administration, Funding acquisition, Conceptualization.

## Ethical approval

This study was reviewed and approved by the Ethics Committee of Zhongshan Hospital, Fudan University (No. B2024-303). For the retrospective clinical data analysis, the requirement for informed consent was waived due to the use of anonymized patient records. All animal experiments were conducted in accordance with ethical guidelines and were approved by the Animal Welfare & Ethics Committee of Zhongshan Hospital (Approval No. 2024-215).

## Declaration of competing interest

The authors declare that they have no known competing financial interests or personal relationships that could have appeared to influence the work reported in this paper.

## Figures and Tables

**figs1:**
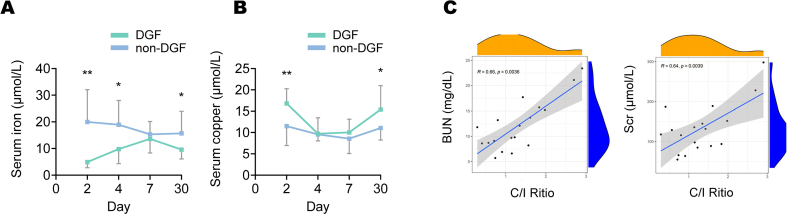


**figs2:**
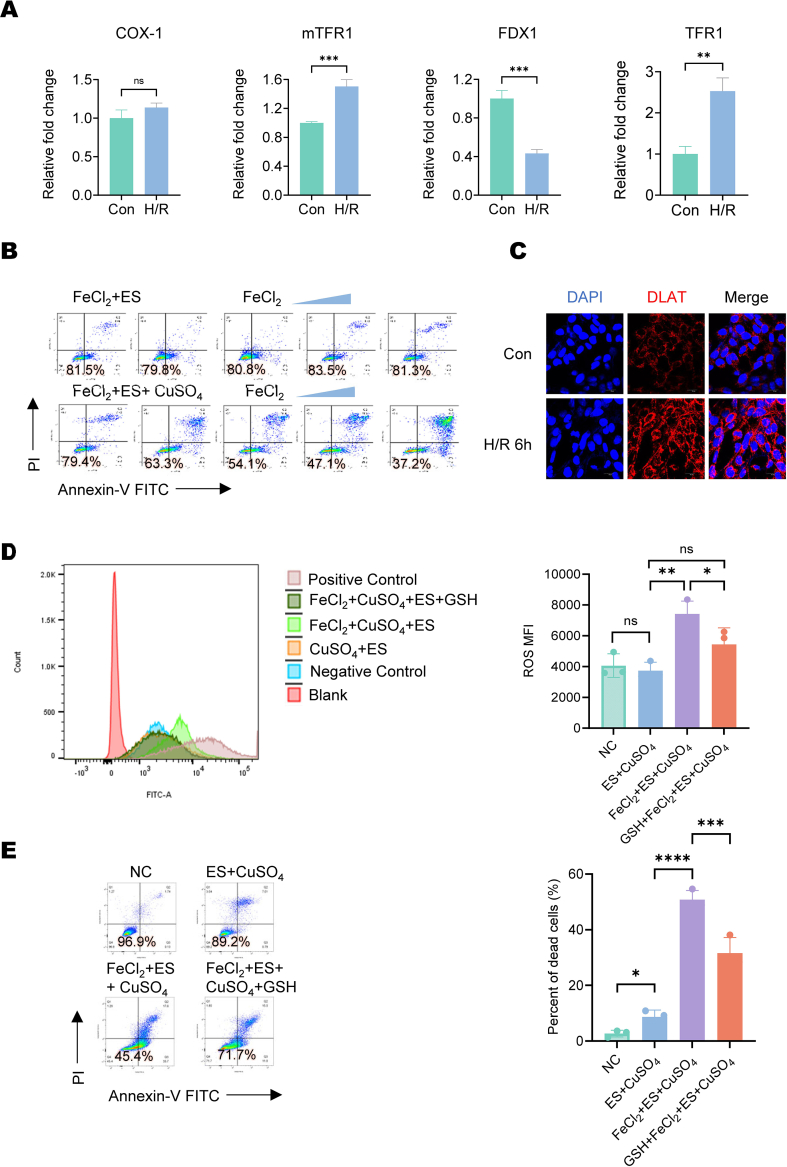


**figs3:**
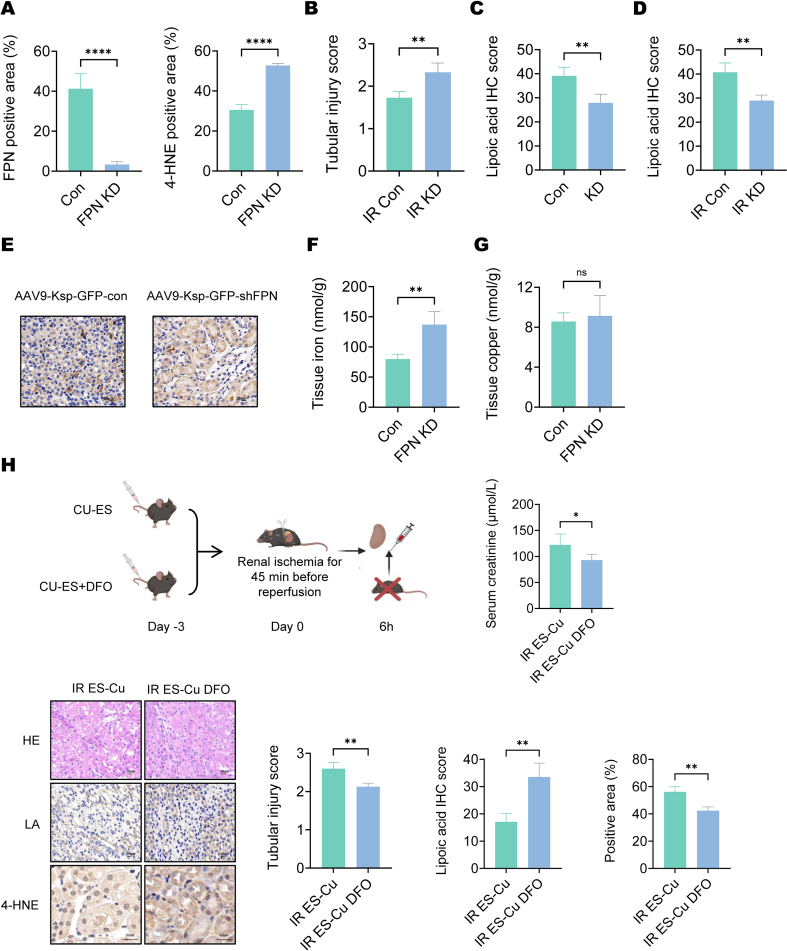


**figs4:**
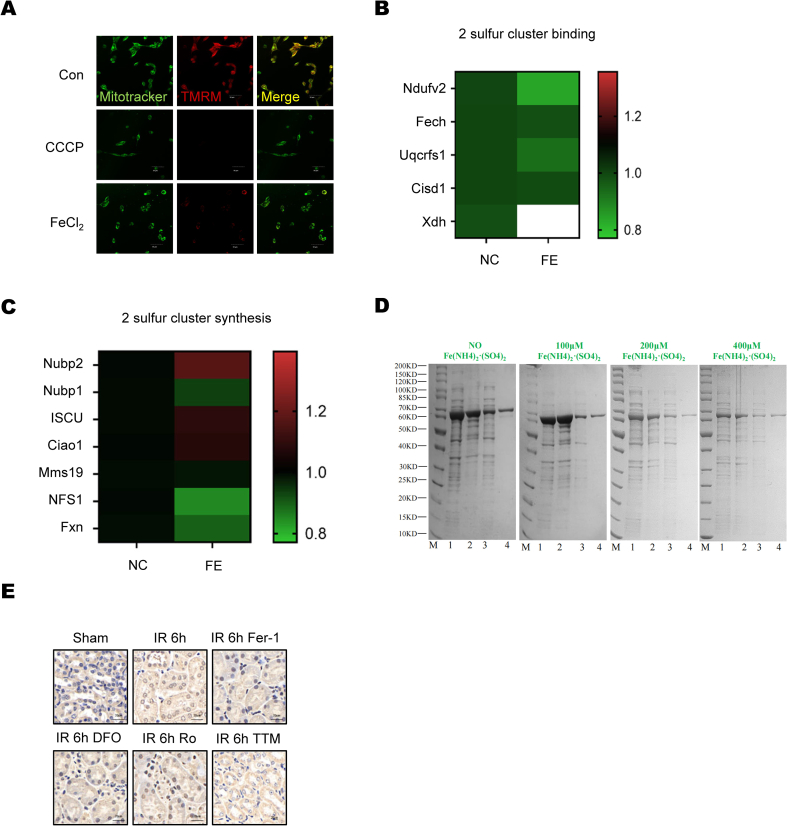


## Data Availability

Data will be made available on request.
