# Supplementary material for: Iron overload exaggerates renal ischemia-reperfusion injury by promoting tubular cuproptosis via interrupting function of LIAS
Source: Redox Biol. 2025 Aug 5;86:103795. doi: 10.1016/j.redox.2025.103795 (PMC12375214; doi:10.1016/j.redox.2025.103795)
Supplement: Multimedia component 1 [file mmc1.docx]

Qpcr primers

| β-actin-m-f | TGTTACCAACTGGGACGACA |
| --- | --- |
| β-actin-m-r | GGGGTGTTGAAGGTCTCAAA |
| kim1-m-f | ACATATCGTGGAATCACAACGAC |
| kim1-m-r | ACTGCTCTTCTGATAGGTGACA |
| ngal-m-f | GCAGGTGGTACGTTGTGGG |
| ngal-m-r | CTCTTGTAGCTCATAGATGGTGC |
| ATP7A-m-F | CCATTGTTACCCTCTTGGTGTGG |
| ATP7A-m-r | CAGAACCGTGATAGAGGCTTGG |
| CTR1-m-f | CGCTACAATTCCATGCCTGTCC |
| CTR1-m-r | GACTACCTGGATGATGTGCAGC |
| fdx1-m-f | CAAGGGGAAAATTGGCGACTC |
| fdx1-m-r | TTGGTCAGACAAACTTGGCAG |
| TFRC-m-f | GTGGAGTATCACTTCCTGTCGC |
| TFRC-m-r | CCCCAGAAGATATGTCGGAAAGG |
| m-fpn-f | ACCAAGGCAAGAGATCAAACC |
| m-fpn-r | AGACACTGCAAAGTGCCACAT |
| m-fth1-f | CAAGTGCGCCAGAACTACCA |
| m-fth1-r | GCCACATCATCTCGGTCAAAA |
| m-ftl1-f | CCATCTGACCAACCTCCGC |
| m-ftl1-r | CGCTCAAAGAGATACTCGCC |

Sg RNA

LIAS-1_F: CACCGCAGGAGTAAGACACTCCACA

LIAS-1_R: AAACTGTGGAGTGTCTTACTCCTGC

LIAS-2_F: CACCGGGCTGGCCCTACCATGATCG

LIAS-2_R: AAACCGATCATGGTAGGGCCAGCCC

Sh RNA

Slc40a1_1_F:GGAUGGGUCUCCUACUAUATT

Slc40a1_1_R:UAUAGUAGGAGACCCAUCCTT

Slc40a1_2_F:GCAGAUUAGCAGACAUGAATT

Slc40a1_2_R:UUCAUGUCUGCUAAUCUGCTT

Slc40a1_3_F:GGCUUUGACUGUAUCACUATT

Slc40a1_3_R:UAGUGAUACAGUCAAAGCCTT
