# Supplementary material for: Iron overload exaggerates renal ischemia-reperfusion injury by promoting tubular cuproptosis via interrupting function of LIAS
Source: Redox Biol. 2025 Aug 5;86:103795. doi: 10.1016/j.redox.2025.103795 (PMC12375214; doi:10.1016/j.redox.2025.103795)

Figure 1

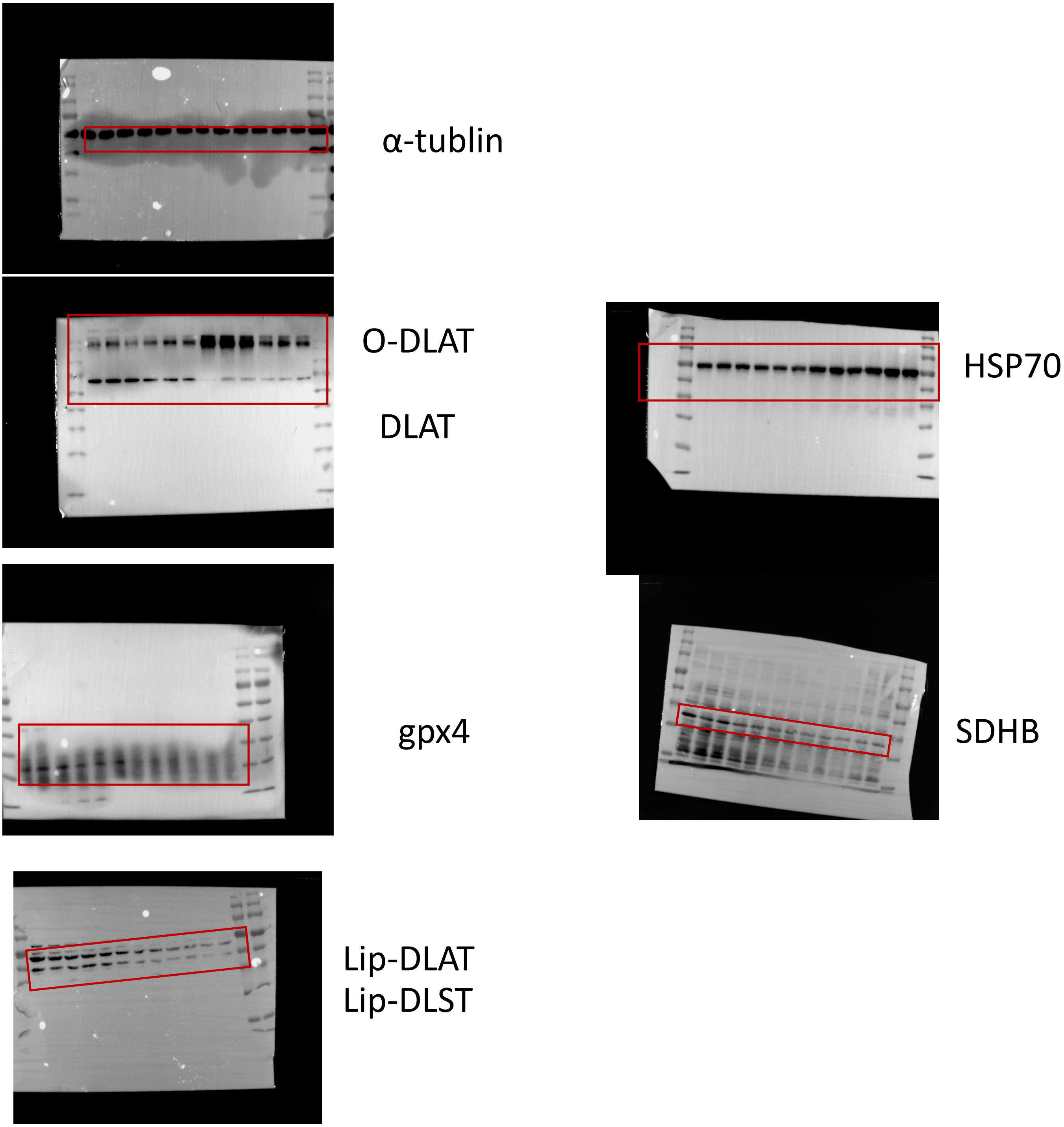

Figure 1

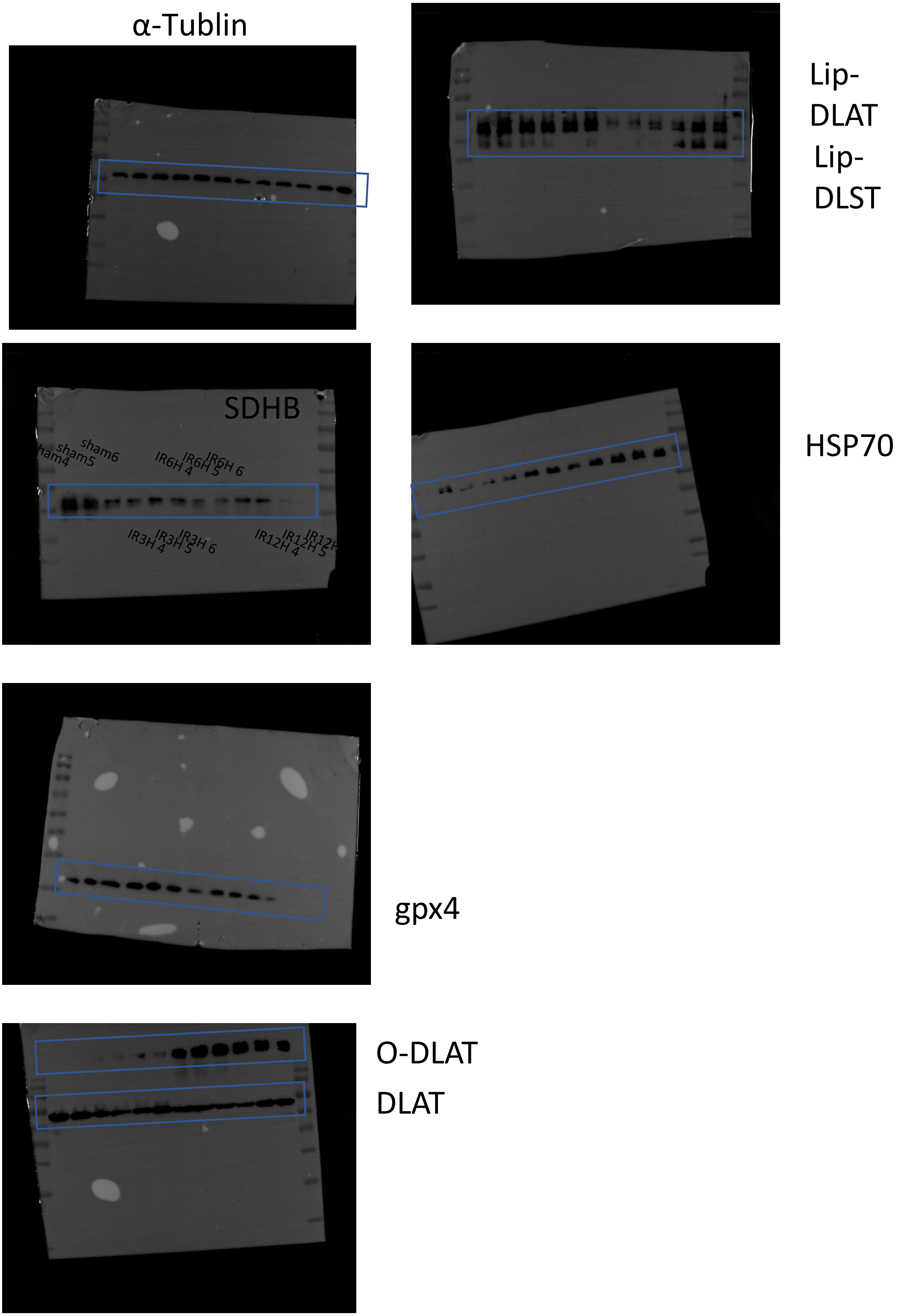
α-Tublin
Lip-
DLAT
Lip-
DLST
SDHB
sham5
sham6
IR6H 4
IR6H 5
IR6H 6
IR3H 4
IR3H 5
IR3H 6
IR12H 4
IR12H 5
HSP70
gpx4
O-DLAT
DLAT

Figure 2

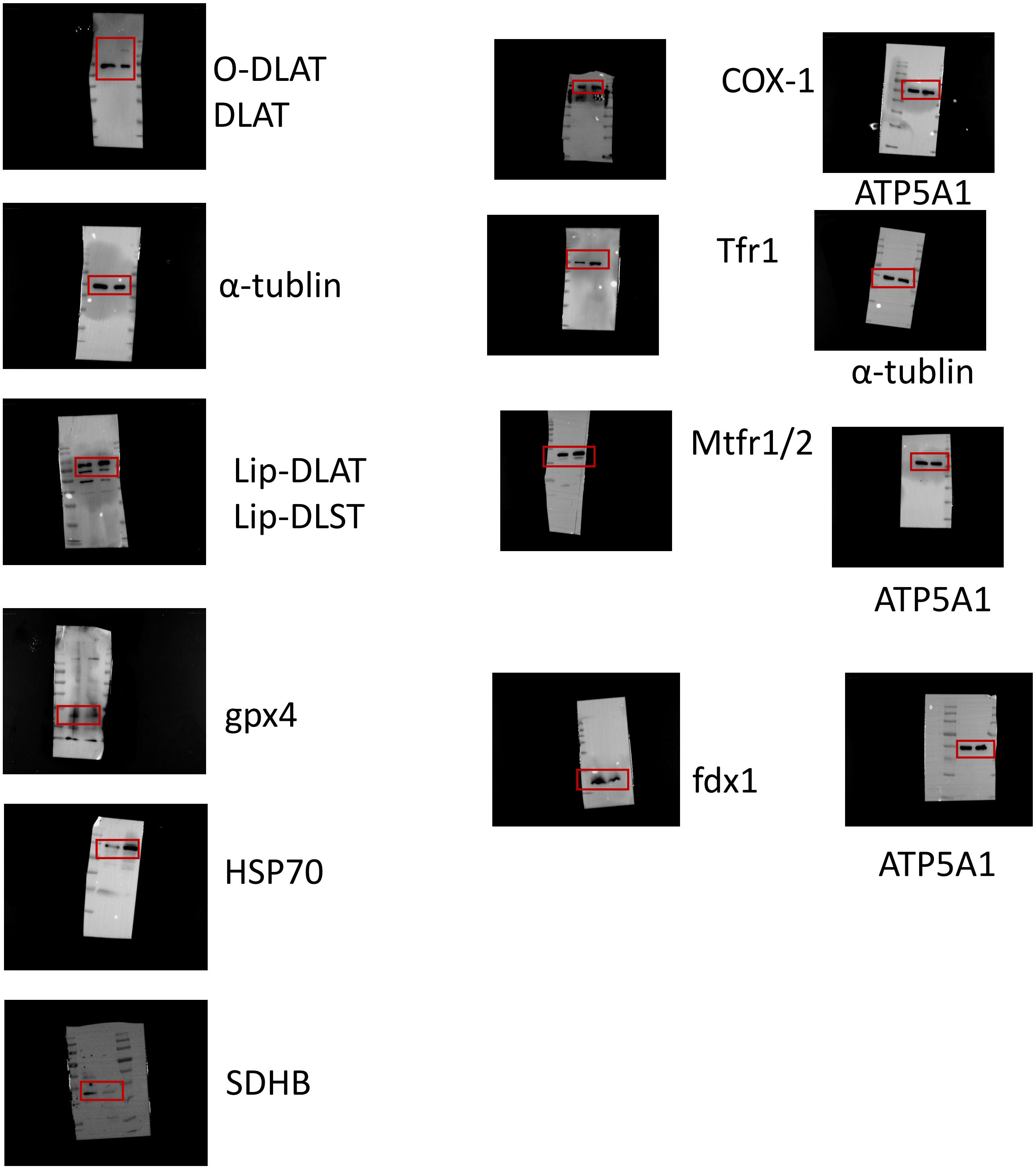
O-DLAT
DLAT
α-tublin
Lip-DLAT
Lip-DLST
gpx4
HSP70
SDHB
COX-1
ATP5A1
Tfr1
α-tublin
Mtfr1/2
ATP5A1
fdx1
ATP5A1

Figure 2

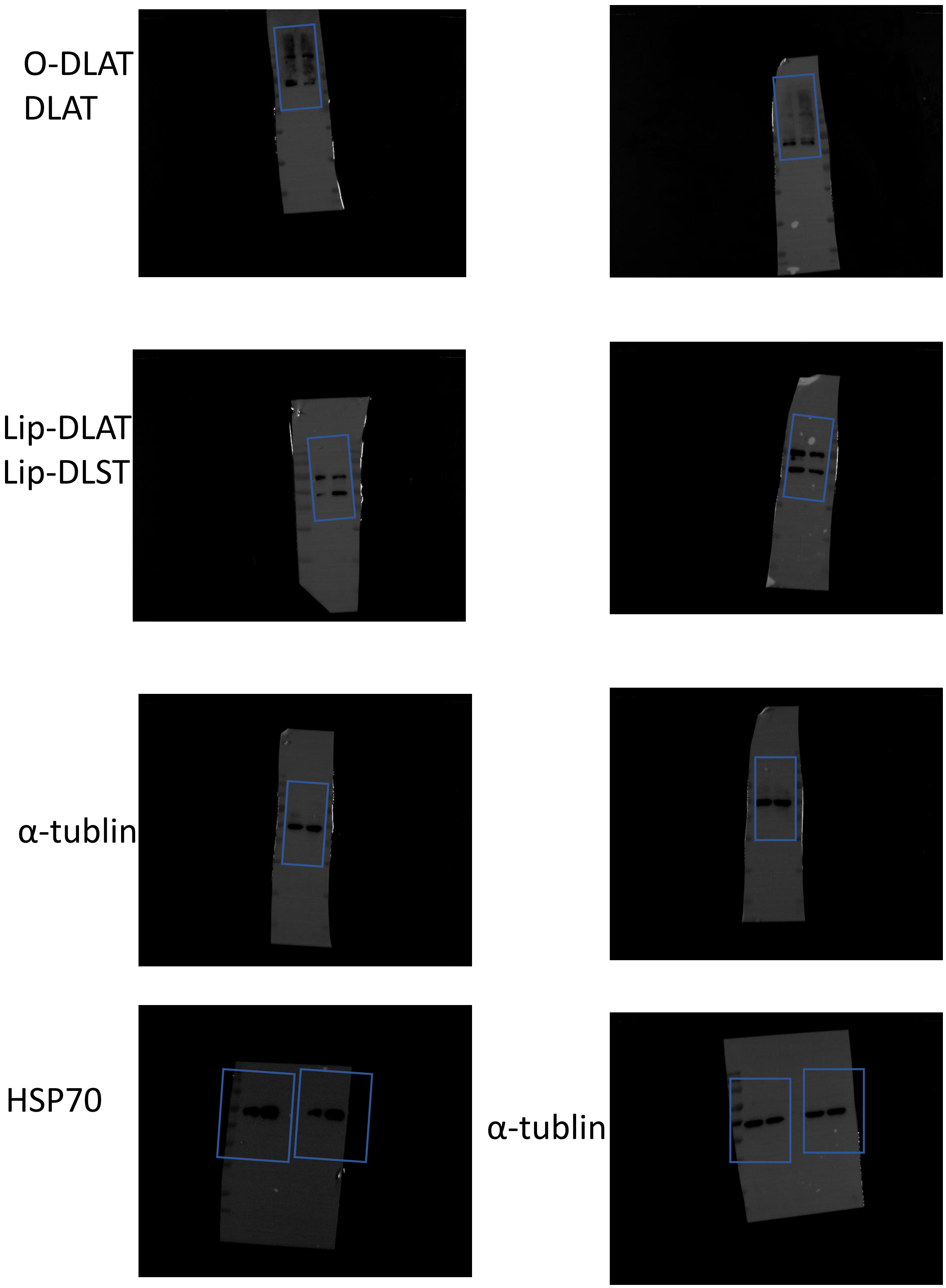

Figure 2

GPX4

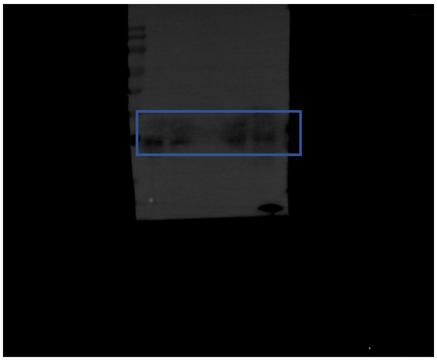

SDHB

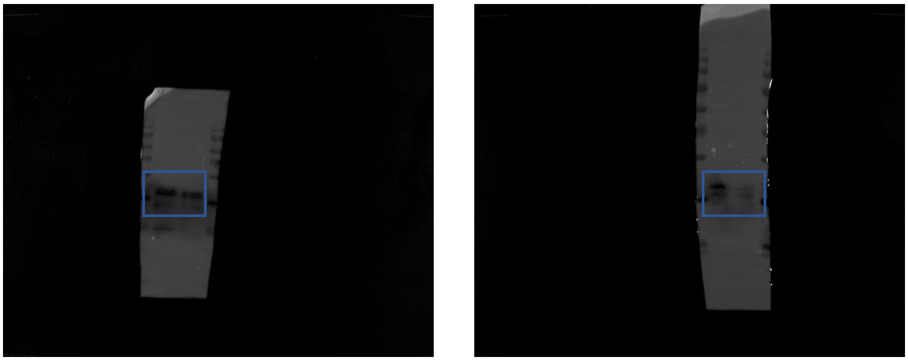

mTFR1/2

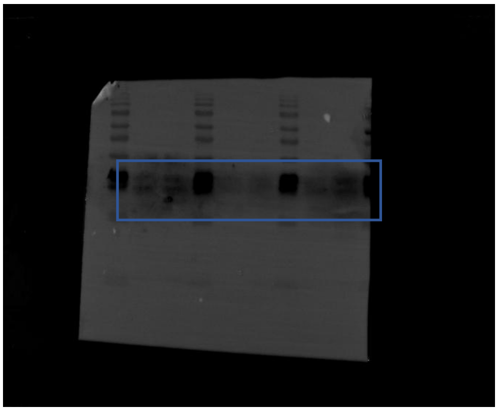

TFR1

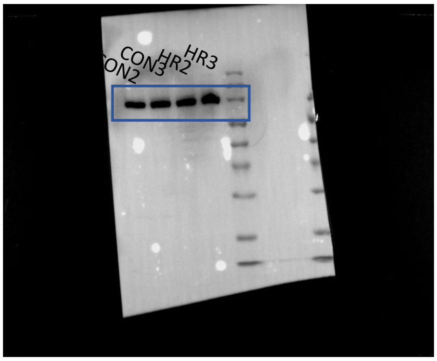

ATP5a1

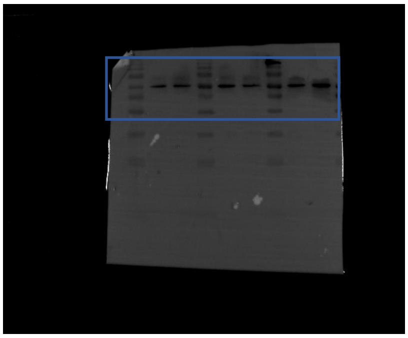

α-tublin

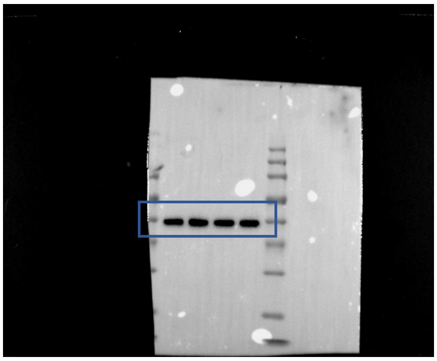

Figure 2

fdx1

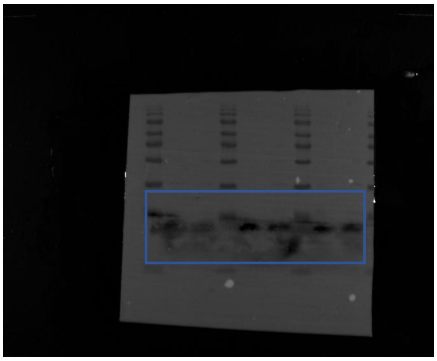

ATP5a1

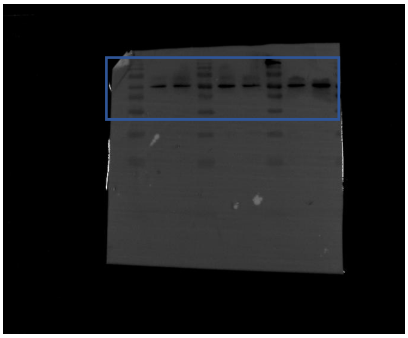

COX-1

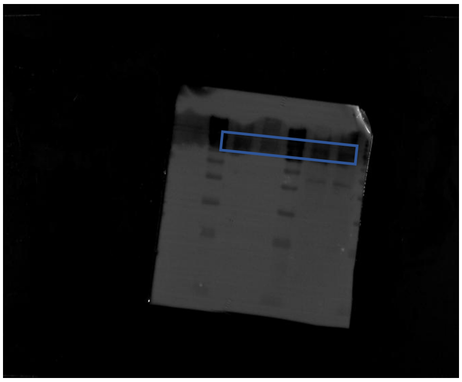

ATP5a1

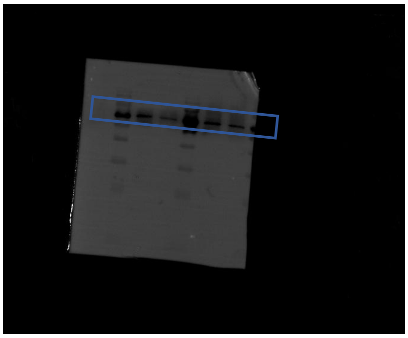

Figure 3

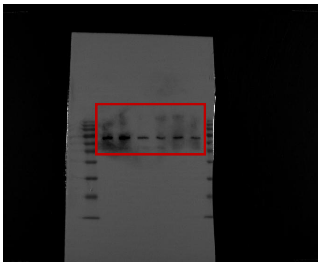

O-DLAT
DLAT

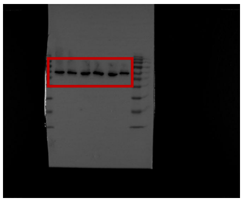

α-Tublin

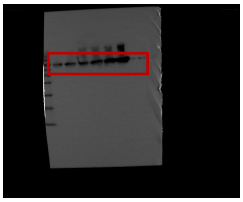

HSP70

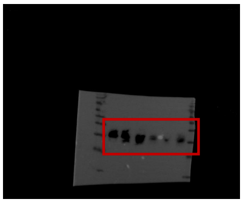

SDHB

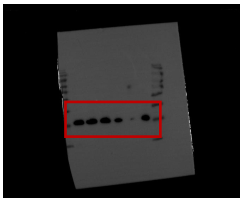

gpx4

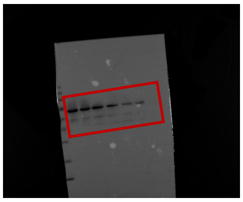

Lip-DLAT
Lip-DLST

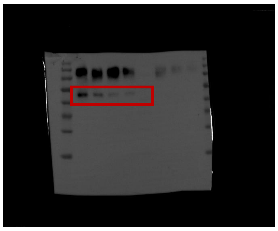

FPN

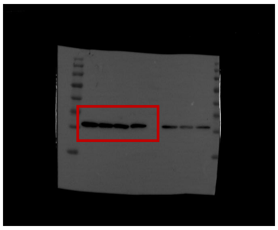

gapdh

Figure 3

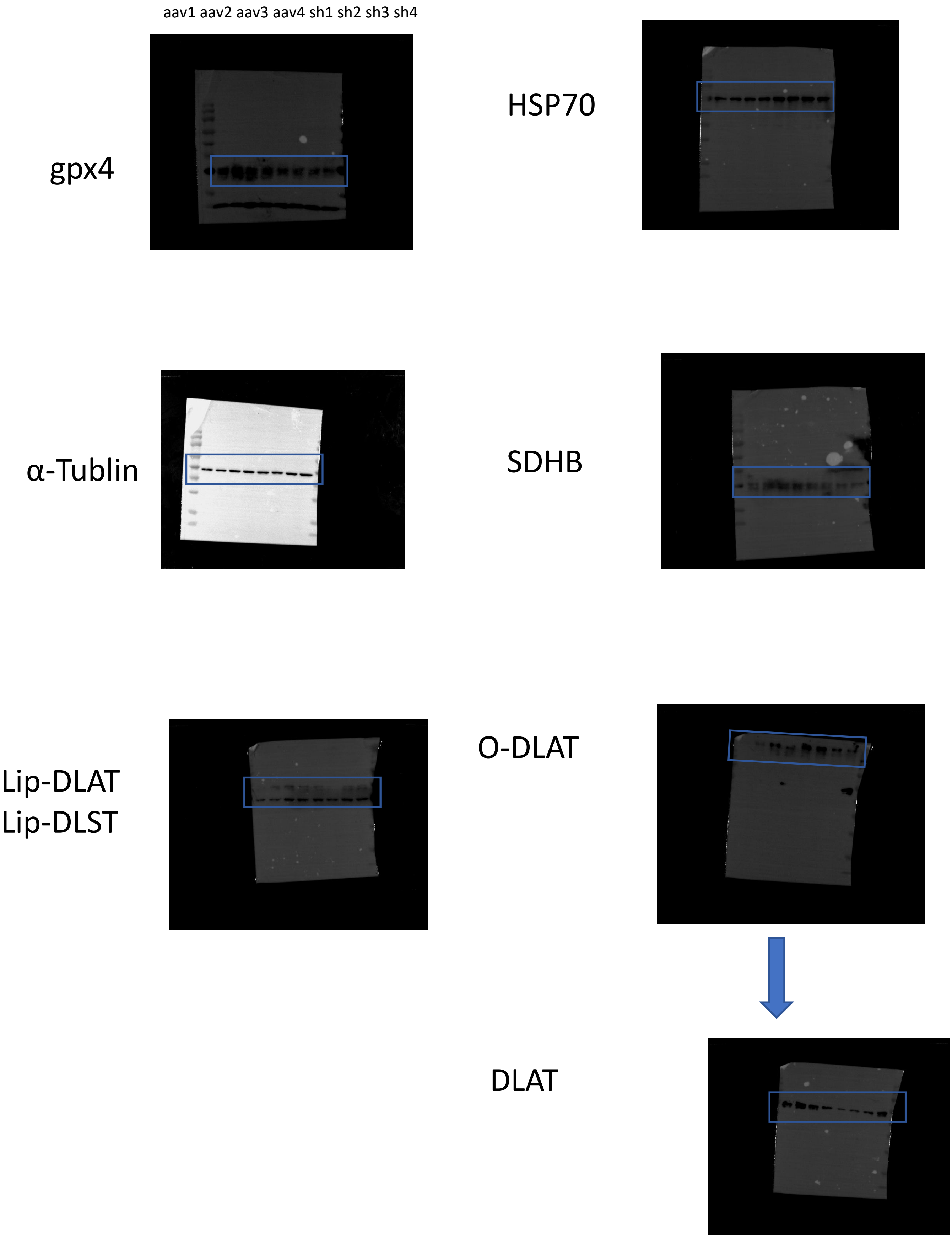
aav1 aav2 aav3 aav4 sh1 sh2 sh3 sh4
gpx4
HSP70
α-Tublin
SDHB
Lip-DLAT
Lip-DLST
O-DLAT
DLAT

Figure 3

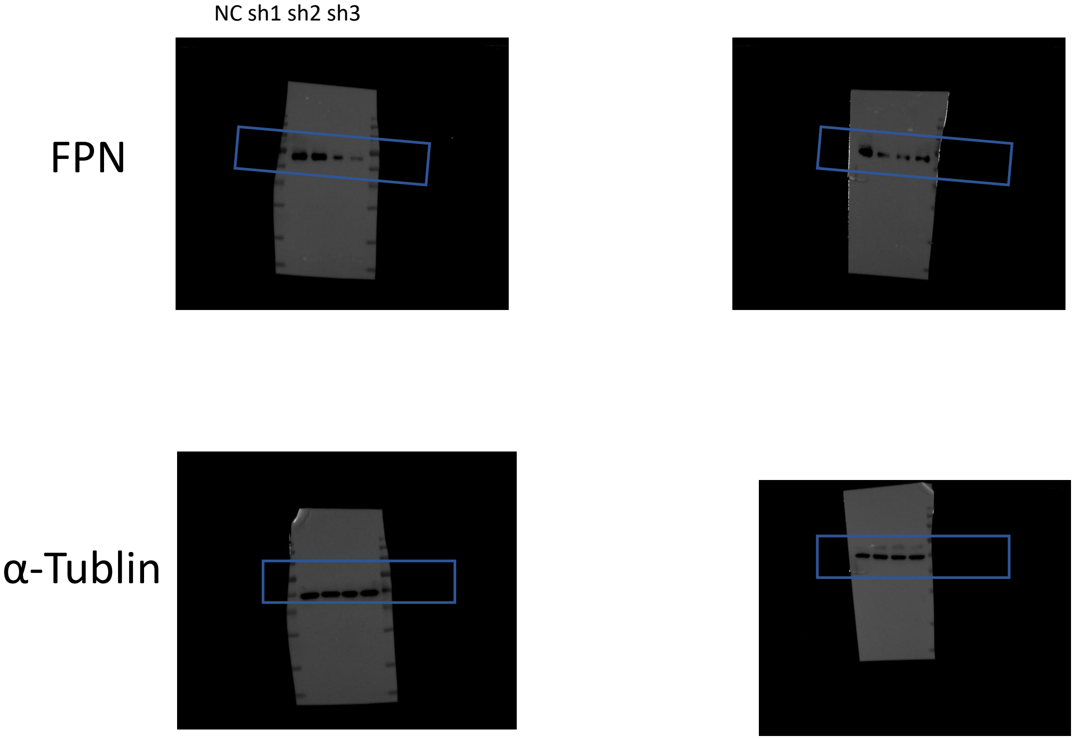

Figure 4

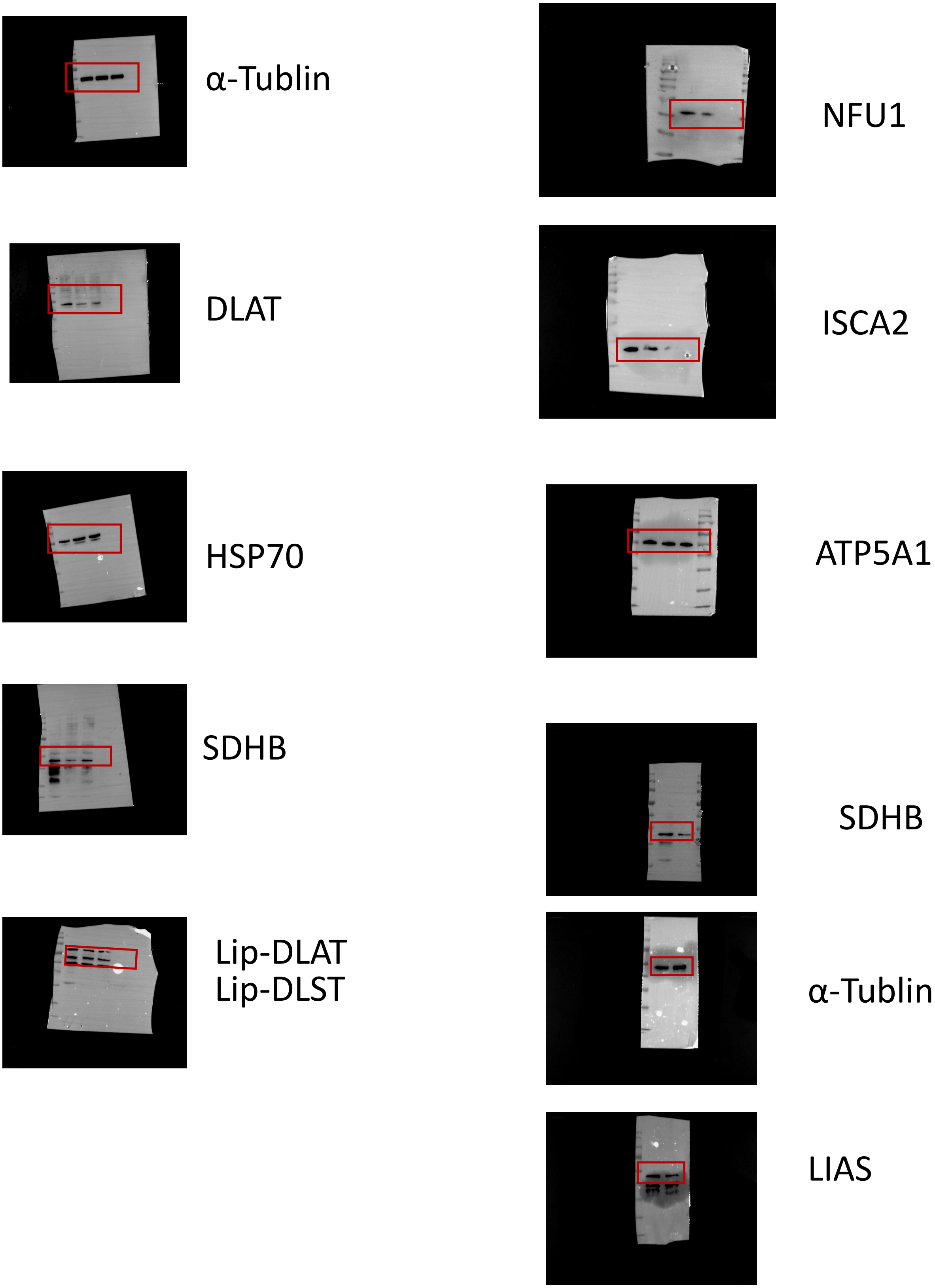
α-Tublin
NFU1
DLAT
ISCA2
HSP70
ATP5A1
SDHB
SDHB
Lip-DLAT
Lip-DLST
α-Tublin
LIAS

Figure 4

HSP70

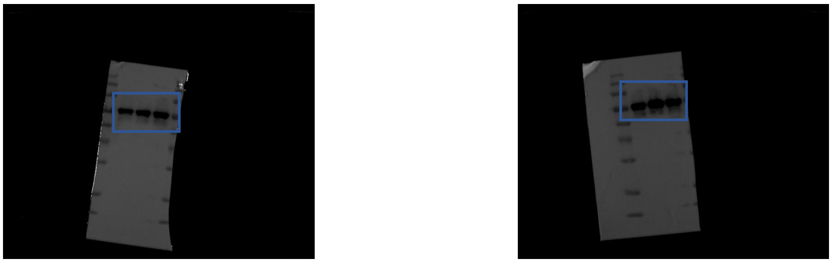

α-Tublin

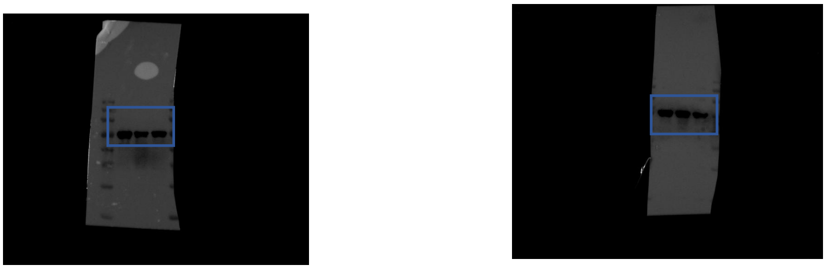

O-DLAT
DLAT

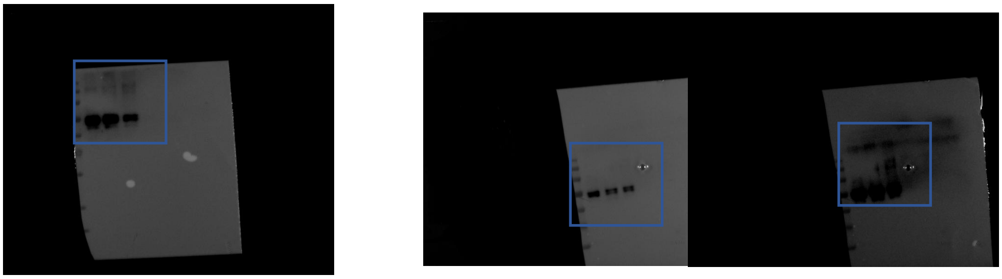

SDHB

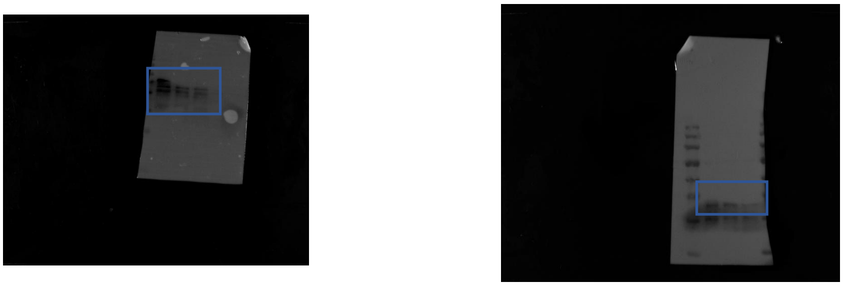

α-Tublin

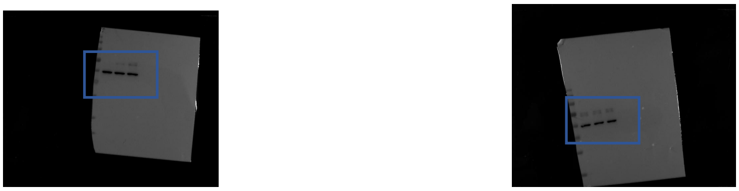

Lip-DLAT
Lip-DLST

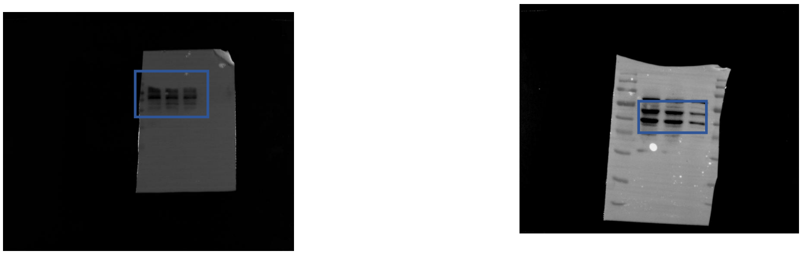

Figure 4

lias

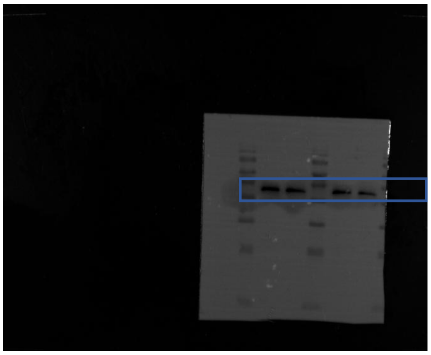

ATP5a1

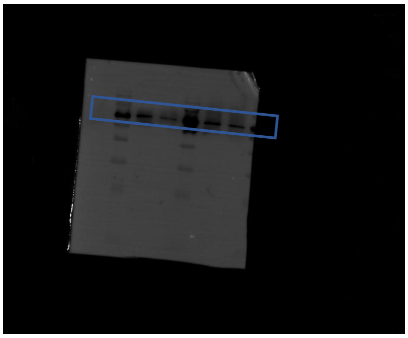

SDHB

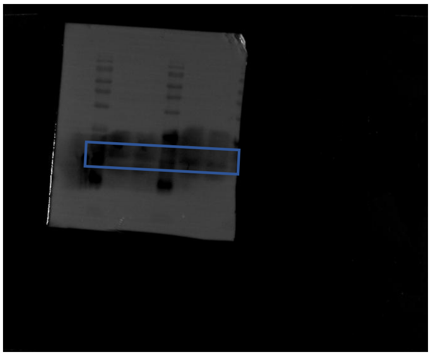

ISCA2

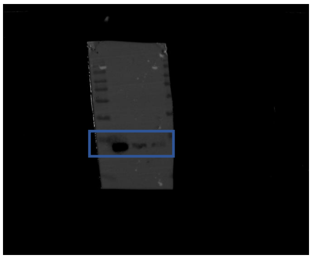

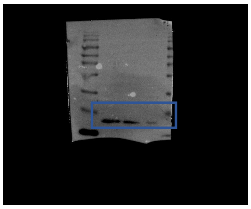

ATP5a1

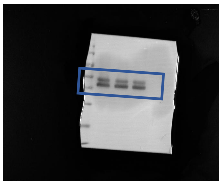

NFU1

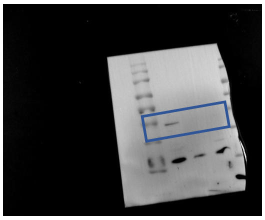

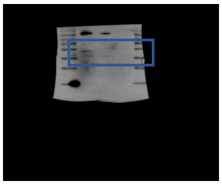

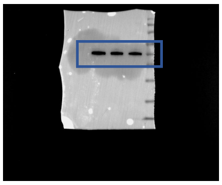

Figure 5

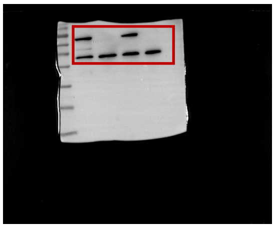

Input
LIAS

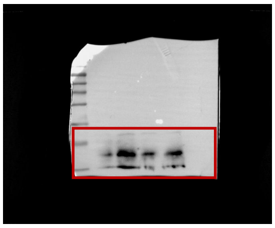

Input
LIAS

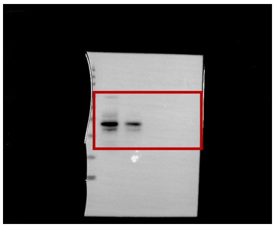

IP
LIAS

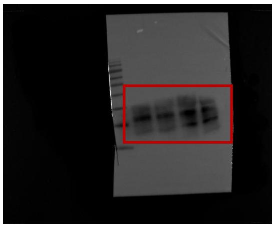

IP
gcsh

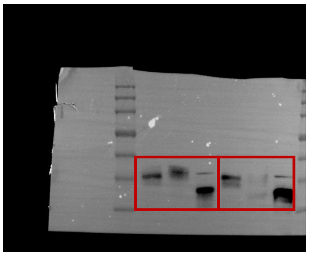

Figure 5C
LIAS

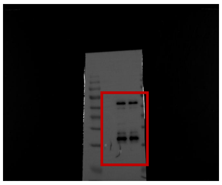

Input
gst

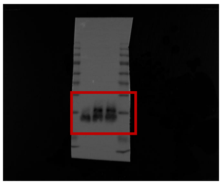

Input
his

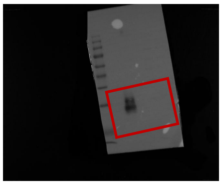

ip
his

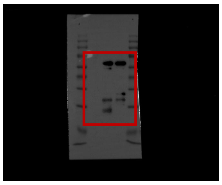

ip
gst

Figure 6

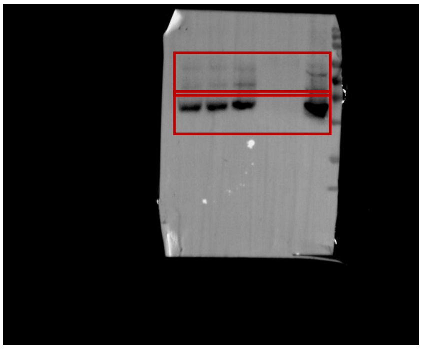

Lip-DLAT
Lip-DLST
LIAS

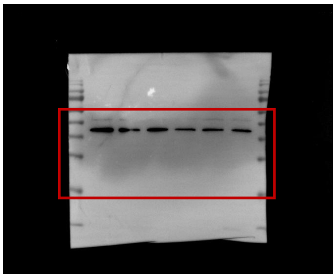

Input
LIAS

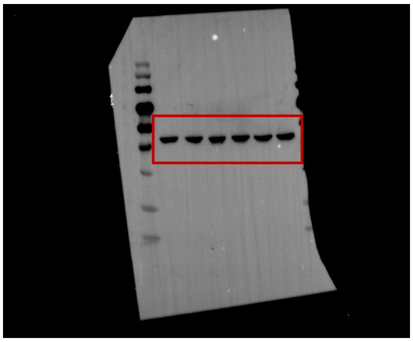

α-Tublin

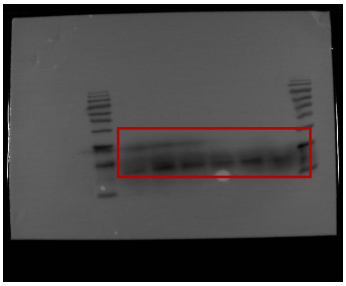

Input
gcsh

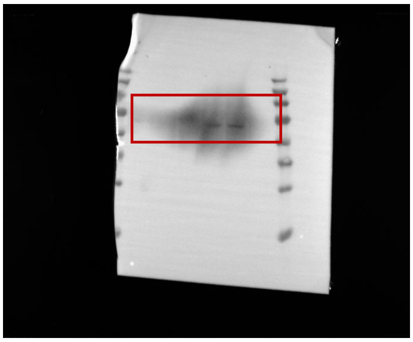

DLAT

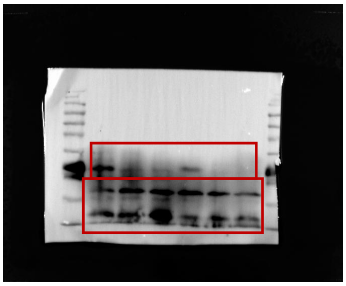

IP
LIAS
GCSH

Figure 6

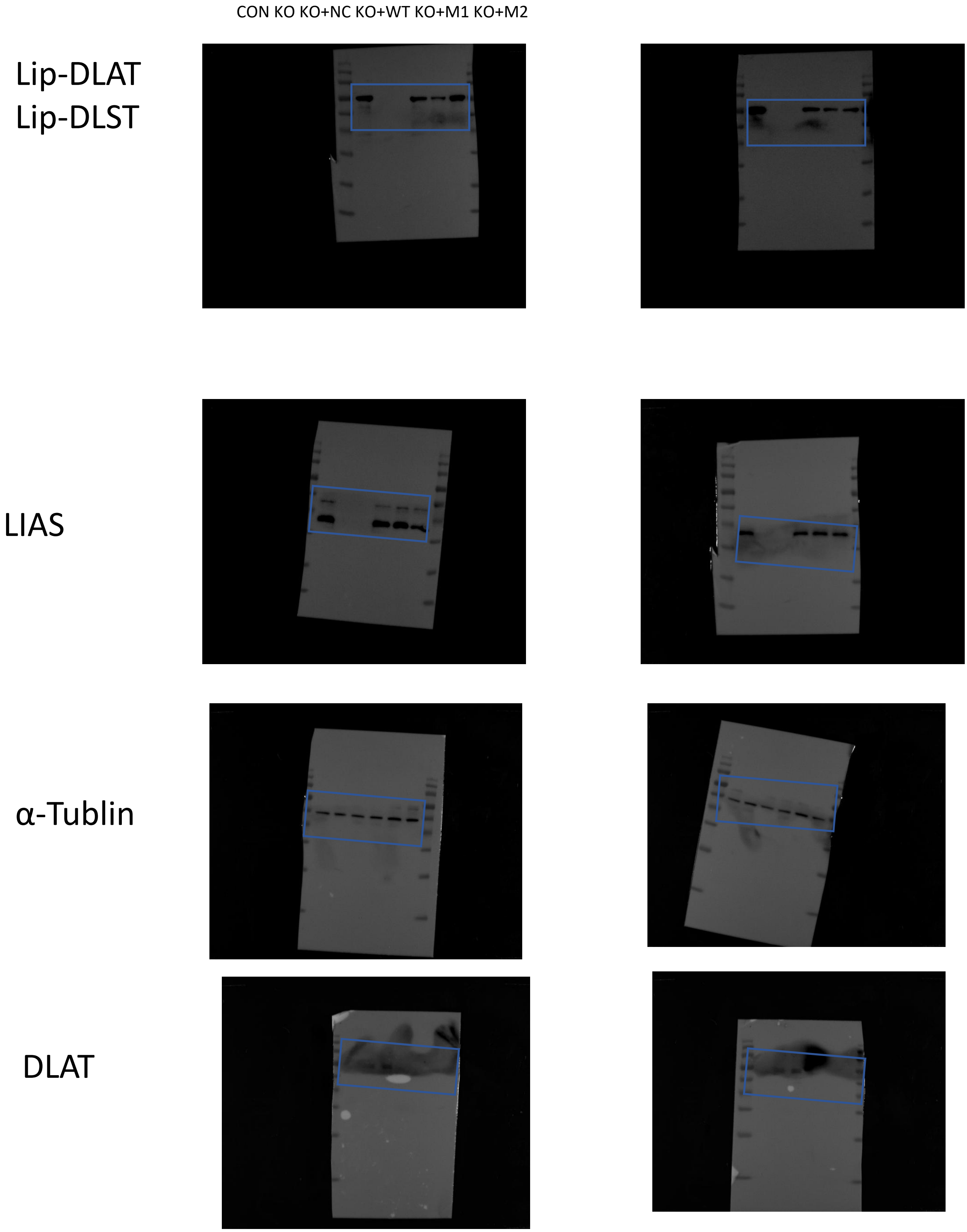

CON KO KO+NC KO+WT KO+M1 KO+M2
Lip-DLAT
Lip-DLST
LIAS
α-Tublin
DLAT

Figure 7

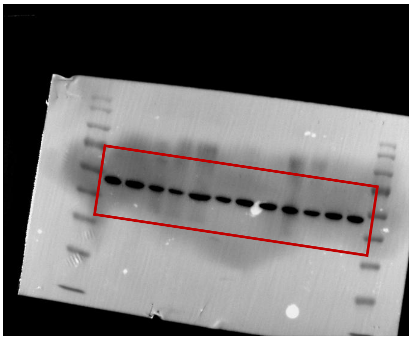

α-Tublin

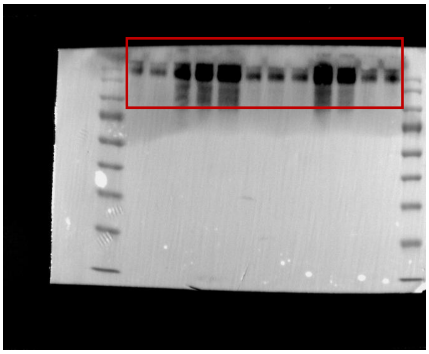

O-DLAT

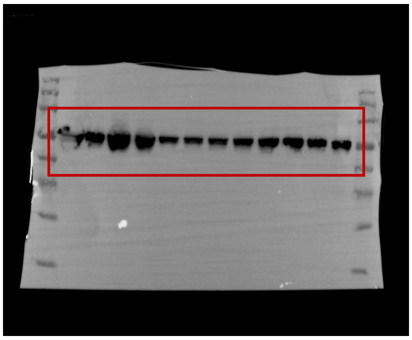

HSP70

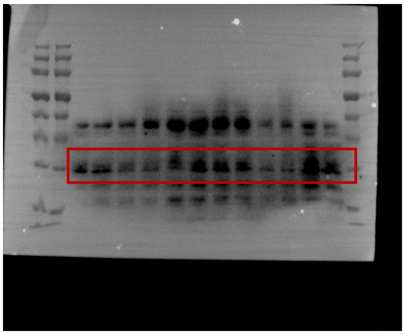

SDHB

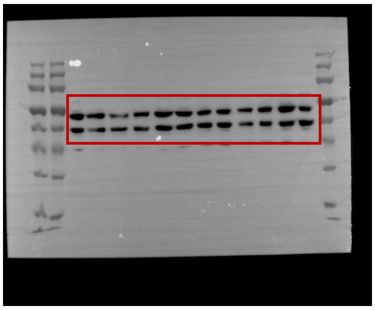

Lip-DLAT
Lip-DLST

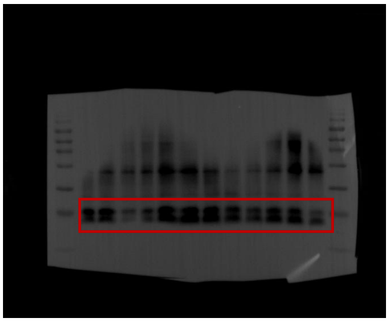

gpx4

Figure 7

CON3 CON4 IR3 IR4 FER-1 3 FER-1 4 DFO3 DFO4 RO3 RO4 TTM3 TTM4

O-DLAT

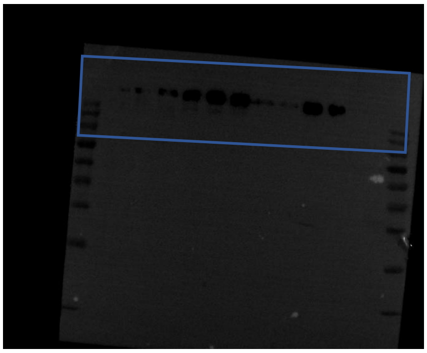

α-Tublin

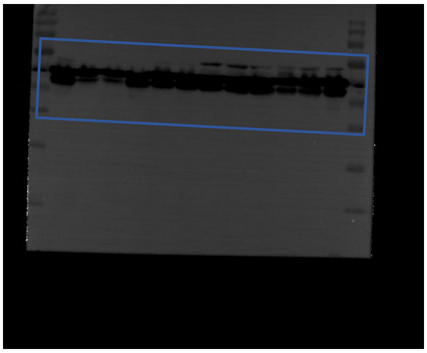

DLAT

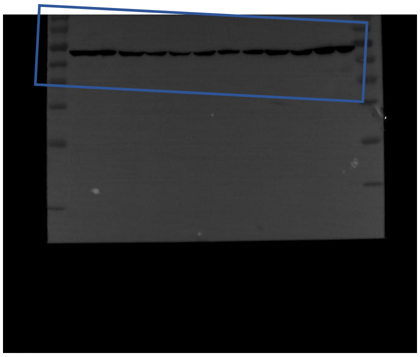

SDHB

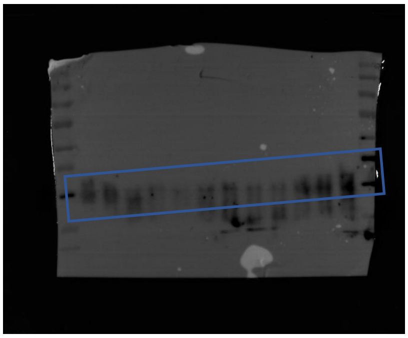

gpx4

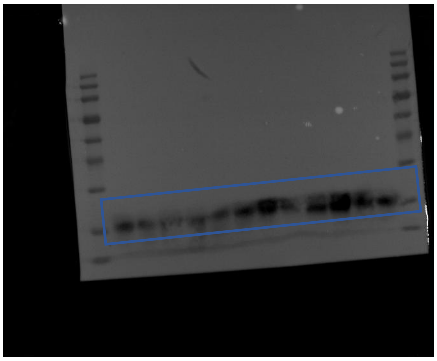

HSP70

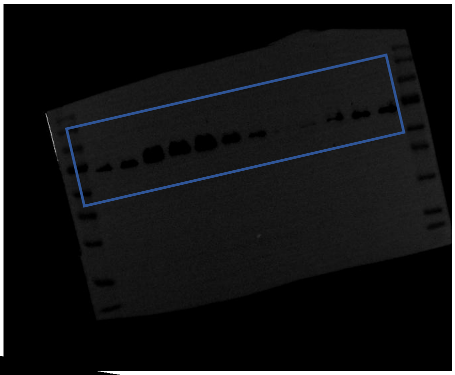

Lip-DLAT
Lip-DLST

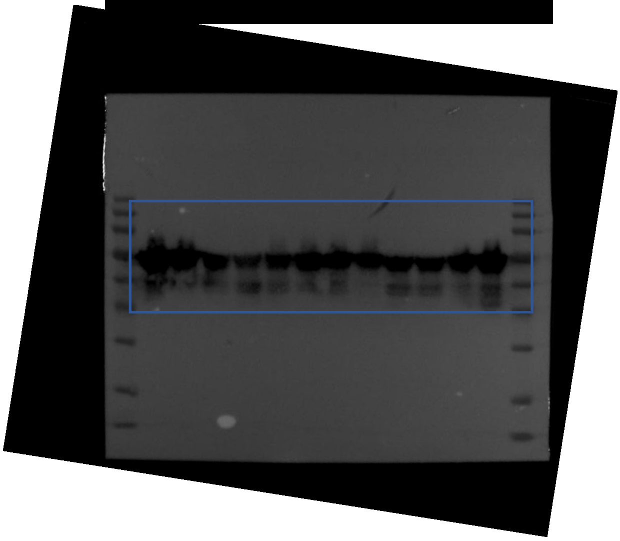

Figure 7

CON5 IR5 FER-1 5 DFO5 RO5 TTM5

O-DLAT

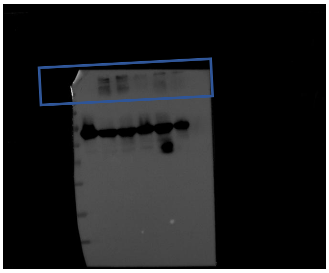

α-Tublin

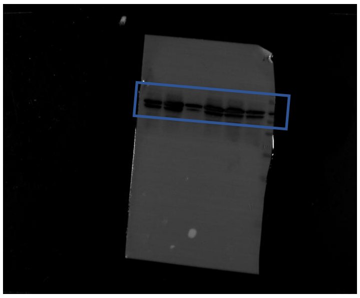

DLAT

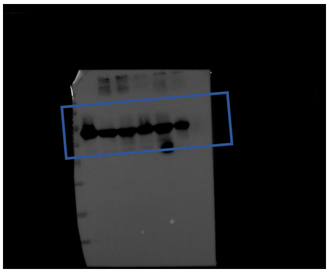

SDHB

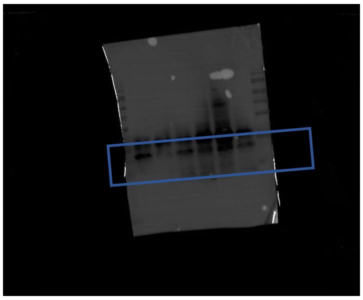

gpx4

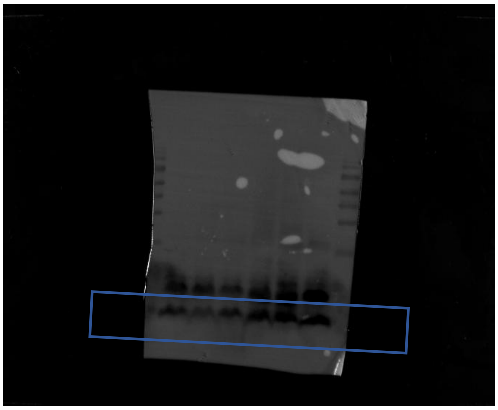

Lip-DLAT
Lip-DLST

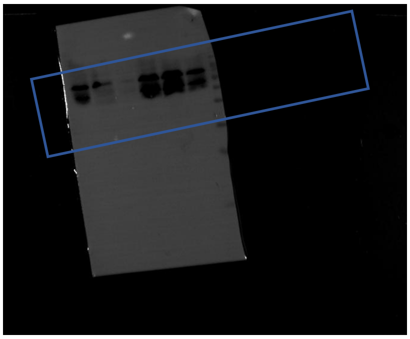

HSP70

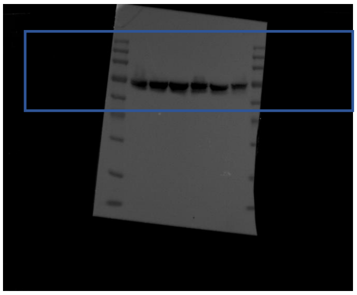

Supplement: Multimedia component 2 [file mmc2.pdf]
